# Confirmation of Connexin45 Underlying Weak Gap Junctional Intercellular Coupling in HeLa Cells

**DOI:** 10.3390/biom10101389

**Published:** 2020-09-29

**Authors:** Eun Ju Choi, Nicolás Palacios-Prado, Juan C. Sáez, Jinu Lee

**Affiliations:** 1 College of Pharmacy, Yonsei Institute of Pharmaceutical Sciences, Yonsei University, Incheon 21983, Korea; yureas@naver.com; 2 Departamento de Fisiología, Pontificia Universidad Católica de Chile, Santiago 6513677, Chile; nicopalacios@uc.cl (N.P.-P.); juancarlos.saez@uv.cl (J.C.S.); 3 Instituto de Neurociencias, Centro Interdisciplinario de Neurociencias de Valparaíso, Universidad de Valparaíso, Valparaíso 2360103, Chile

**Keywords:** gap junction, iodide-yellow fluorescent protein GJIC assay, HeLa cell, CRISPR/Cas9

## Abstract

Gap junctions (GJs) are intercellular channels that connect adjacent cells electrically and metabolically. The iodide-yellow fluorescent protein (I-YFP) gap junctional intercellular communication (GJIC) assay is a recently developed method with high sensitivity. HeLa cells have been widely used as GJ-deficient cells for GJ-related research. Herein, we present evidence showing that HeLa cells have functional GJs comprising connexin (Cx) 45 using the I-YFP GJ assay and CRISPR/Cas9 system. We conducted the I-YFP GJIC assay in HeLa cells, which revealed a weak level of GJIC that could not be detected by the Lucifer yellow scrape-loading assay. The mRNA expression of *GJB5* (Cx31.1), *GJA1* (Cx43), and *GJC1* (Cx45) was detected in HeLa cells by RT-PCR analysis. Knocking out *GJC1* (Cx45) abolished GJIC, as analyzed by the I-YFP assay and dual whole-cell patch-clamp assay. These results suggest that HeLa cells express Cx45-based GJs and that the I-YFP GJIC assay can be used for cells with weak GJIC, such as Cx45-expressing HeLa cells. Further, *GJC1* (Cx45)-knockout HeLa cells are more suitable as a GJ-null cell model for transfection experiments than wild-type HeLa cells. This experimental design was successfully applied to knock out Cx43 expression and GJIC in A549 lung cancer cells and can thus be used to identify major Cxs in other cell types and to establish GJ assay systems for different Cxs.

## 1. Introduction

Gap junctions (GJs) are intercellular channels located on cell-to-cell interfaces through which ions and metabolites less than about 1 kDa can diffuse between cells [1]. Six connexins (Cxs) oligomerize to form a hemichannel (HC), which can form a GJ by docking to another HC on a neighboring cell surface [2]. The Cx gene family consists of 21 members in the human genome, and it is divided into five subfamilies (*GJA*, *GJB*, *GJC*, *GJD*, and *GJE*) according to their sequence homology [3]. Their proteins are named based on the approximate molecular weight in kDa, i.e., Cx43 for *GJA1*. *GJA1* (Cx43) and *GJB2* (Cx26) have been the most intensively studied [4].

Gap junctional intercellular communication (GJIC) significantly contributes to normal physiology, and therefore mutations in Cx genes may cause different diseases [5] such as developmental defects (*GJA1*), skin diseases (*GJA1, GJB2, GJB3, GJB4*, and *GJB6*), hearing loss (*GJB2, GJB3, GJB4,* and *GJB6*), and cataracts (*GJA3* and *GJA8*). GJs also play an important role in pathological conditions, including ischemia/reperfusion injury [6,7,8], epilepsy [9,10], inflammation [11,12], and neurodegenerations [13].

GJIC measurement is essential in the related studies. The most commonly used methods include permeability assays (e.g., dye transfer technique) and conductance assays (electrophysiology) [14]. The former methods are based on the diffusion of membrane impermeant fluorescent dyes such as Lucifer yellow (LY) through GJs [15,16], and the latter methods measure transjunctional current to be transferred between two cells connected via GJs using dual whole-cell patch clamp technique [17]. Recently, the iodide-yellow fluorescent protein (I-YFP) GJIC assay was developed as a high-throughput screening (HTS)-compatible method [18]. Iodide movement through GJs is measured using YFP^QL^ quenching, which reflects GJIC activity. Despite a recent advance [19], this assay is advantageous because it is simple, fast, non-laborious, and inexpensive compared to the conventional methods mentioned above. Another merit of this assay is the potential to enhance sensitivity by extending the assay time.

HeLa cells are human cervical cancer cells, which are the oldest and the most commonly used cell lines in cell-based scientific research. Scientists in the GJ field have also utilized these cells as “GJ-null cells” to study specific Cxs because the cells showed no dye transfer and weak electrical coupling [20,21] and because functional GJs are easily formed with many different exogenously expressed Cxs [20,21,22,23]. However, by enhancing sensitivity with I-YFP GJIC assay, we were able to show weak but measurable levels of GJIC in monolayers of HeLa cells. We also used dual whole-cell patch-clamp and the CRISPR/Cas9 knockout (KO) system to show that HeLa cells express functional GJs composed of Cx45. This strategy was also successfully applied to show that Cx43-based GJs are expressed in A549 lung cancer cells.

## 2. Materials and Methods

### 2.1. Reagent

The antibodies used in this study were as follows: mouse monoclonal anti-Cx45 (MAB3100, Merck Millipore, Billerica, MA, USA), mouse monoclonal anti-Cx43 (C13720, BD Transduction Laboratories™, San Joes, CA, USA), goat polyclonal anti-actin (C-11, Santa Cruz Biotechnology, Santa Cruz, CA, USA), and goat anti-mouse IgG (H + L) HRP-conjugated (62-6520, Thermo Fisher Scientific, Rockford, IL, USA) antibodies. RNeasy^®^ Mini Kit (74104, Qiagen, Hilden, Germany), PrimeScript TM 1st strand cDNA kit (6110A, Takara Bio, Shiga, Japan), Taq polymerase PCR kit (DT95-E500, Solgent, Daejeon, Korea), tissue DNA Purification Kit (CME0112, Cosmogenetech, Daegu, Korea), T7 endonuclease 1 (T7E1) (M0302L, New England Biolabs, Ipswich, MA, USA), and LY (L0144, Sigma-Aldrich, St Louis, MO, USA) were purchased from local vendors.

### 2.2. Cell Culture

HEK293T (ATCC, Manassas, VA, USA), HeLa (ATCC) and engineered HeLa cells were cultured in Dulbecco’s Modified Eagle medium (DMEM) containing 100 IU/mL penicillin, 100 mg/mL streptomycin, and 10% fetal bovine serum. RPMI medium 1640 was used for A549 (ATCC) and its derivatives. Cells were maintained at 37 °C in a humidified atmosphere with 95% air and 5% CO_2_. The identity of HeLa and A549 cells and their derivatives was confirmed by STR profiling (File S1), and phase-contrast cell images (File S2).

### 2.3. Constructs

To generate lentiviral plasmids expressing YPF^QL^ or SLC26A4 under cytomegalovirus (CMV) promoter, two empty vectors, pLVX-CMV-IRES-Blasticidin (pLVX-CIBla) and pLVX-CMV-IRES-Hygromycin (pLVX-CIH), were generated by modifying pLVX-EF1α-IRES-Puro (631988, Takara Bio). Next, pLVX-CIBla-YFP^QL^ and pLVX-CIH-SLC26A4 were constructed by inserting a DNA fragment encoding the respective CDS. YFP^QL^ is a YFP variant whose fluorescence is sensitively quenched by iodides [24].

The oligonucleotides in Table 1 were annealed and inserted at the BsmBI site of the LentiCRISPRv2 plasmid (Addgene 52961). The resulting plasmids were used to knock out *GJB2* (Cx26), *GJB5* (Cx31.1), *GJA1* (Cx43), and *GJC1* (Cx45).

### 2.4. Lentiviral Production and Transduction

HEK293T cells were plated on 6-well plates at a density of 4 × 10^5^ cells/well and grown for 24 h. The lentiviral plasmid, packaging plasmid (psPAX2, Addgene 12260), and envelope plasmid (pMD2.G, Addgene 12259) were combined at a ratio of 4:3:1. The 3-μg mixture was transfected into HEK293T cells with polyethylenimine (23966-1, Polysciences, Inc., Philadelphia, PA, USA) for 15 h. The cells were refreshed using 2 mL of fresh medium for each well and cultivated for another 48 h. The media containing the lentivirus were harvested by centrifugation at 3000× *g* for 5 min before filtering through 0.4 μm and storing at −80 °C.

To establish stable cell lines, cells were seeded on 6-well plates at 50% confluence 24 h before their transduction with 500 μL of medium containing lentivirus mixed with 1.5 mL of fresh growth medium for 15 h. Next, fresh growth medium was added to the cells, followed by incubation for 48 h before selecting the positive transformants with 2 μg/mL puromycin. Several hundred clones were obtained and pooled for all stable cells used in this study, which precluded clone-specific phenomena.

### 2.5. I-YFP GJIC Assay

Donor and acceptor cells were mixed at a ratio of 4:1 and plated on 96-well plates. After 24 h of cultivation, the cells were washed with 1× PBS and incubated with 100 μL of C-solution (10 mM HEPES, pH 7.4, 140 mM NaCl, 10 mM glucose, 5 mM KCl, 1 mM MgCl_2_, and 1 mM CaCl_2_). The GJIC assay was conducted well by well. The fluorescence of a well was measured using a FLUOstar microplate reader (BMG Labtech, Ortenberg, Germany) for 160 (HeLa) or 30 s (A549) with a 2- (HeLa) or 0.5-s (A549) interval. One second after the first measurement, 100 μL of I-solution (10 mM HEPES, pH 7.4, 140 mM NaI, 10 mM glucose, 5 mM KCl, 1 mM MgCl_2_, and 1 mM CaCl_2_) were added via the automated injector in the plate reader. The percentage of YFP quenching was calculated using Equation (1). The GJIC activity was defined as the difference between YFP quenching of acceptor cells mixed with non-transfected (WT) cells and that of mixed acceptor and donor cell culture at the end of each assay. Relative GJIC activity was calculated as a percentage of GJIC activity compared to the control group.

(1) $$\mathrm{YFP}\;\mathrm{quenching}\;\left(\%\right)=\left(1-\frac{\mathrm{YFP}\;\mathrm{Fluorescence}}{\mathrm{YFP}\;\mathrm{Fluorescence}\;\mathrm{at}\;4\;s}\right)\;\times \;100$$

### 2.6. Reverse Transcription-Polymerase Chain Reaction (RT-PCR)

We performed RT-PCR analysis to investigate the mRNA expression of *GJB2* (Cx26), *GJB4* (Cx31.1), *GJA1* (Cx43), and *GJC1* (Cx45) in HeLa cells or A549 cells. Total RNA was prepared from indicated cells using the RNeasy^®^ Mini Kit. The cDNAs were synthesized using the PrimeScript TM 1st strand cDNA kit with oligo-dT primers. PCR was conducted using the Solgent PCR kit and appropriate primers from each cDNA template according to the manufacturer’s indication. Next, the PCR products were analyzed using 2% agarose gel electrophoresis.

### 2.7. T7E1 Assay

Once CRISPR/Cas9 induces a double-strand break in genomic DNA at specific sites guided by sgRNA, random insertions or deletions are generated during the cellular DNA repair process. Thus, gene modification by the Cas9 system can be assessed with the extent of polymorphism at the target site. Here, T7E1, which cleaves dsDNA at mismatch sites, was used to assess gRNA efficiency [25]. The genomic DNA fragments, including gRNA targets, were PCR-amplified with appropriate primers using a standard PCR procedure. The PCR products were diluted in 1× NEBuffer2 (New England Biolabs) and re-annealed by incubating at 95 °C for 5 min before gradually reducing the temperature to 25 °C at a rate of 0.1 °C/s. The heteroduplex amplicons were subsequently treated with T7E1 for 30 min at 37 °C and analyzed using 2% agarose gel electrophoresis. Band intensity was analyzed using ImageJ software. The percentage (%) of gene modification was calculated as follows:

(2) $$\%\;\mathrm{Gene}\;\mathrm{modification}=100\;\times \;(1-{\left(1-\mathrm{faction}\;\mathrm{cleaved}\;\right)}^{\frac{1}{2}})$$

### 2.8. Immunoblot Analysis

Cells confluent on 6-well plates were washed with 2 mL 1× PBS and lysed with 200 μL of 1× PBS containing 1% Triton X-100 and Complete Protease inhibitor cocktail (25178600, Roche, Basel, Switzerland). The protein concentration was determined using the BCA assay. Equal amount of each protein sample (20 μg) were separated using sodium dodecyl sulfate–polyacrylamide gel electrophoresis (SDS-PAGE) and transferred to nitrocellulose membranes (10600002; GE healthcare life science, PA, USA). Cx45, Cx43, and actin were visualized using corresponding primary antibodies and HRP-conjugated secondary antibodies.

### 2.9. Measurement of Junctional Conductance (Gj) and its Dependence on Junctional Potential (Vj)

Electrophysiology experiments were performed in a modified Krebs–Ringer (MKR) solution containing (in mM): NaCl, 140; CsCl, 5.4; CaCl_2_, 1.8; MgCl_2_, 1; BaCl_2_, 2; and HEPES, 5 (pH 7.4). Electrodes were filled with a pipette solution containing (in mM): CsCl, 130; NaAsp, 10; CaCl_2_, 0.26; MgCl_2_, 1; EGTA, 2; TEACl, 7; and HEPES, 5 (pH 7.2). Cells grown on glass coverslips (80–100% confluence) were transferred to an experimental chamber mounted on an Olympus X-70 microscope and perfused with MRK solution at 30 °C. Experiments were performed in spontaneously formed cell pairs expressing endogenous GJ channels. 

The dual whole-cell patch-clamp technique was used to measure *G_j_*. Briefly, a Multiclamp 200b amplifier with two microelectrodes (resistance of 3–6 MΩ) was used to measure *G_j_*, where V_1_, V_2_, I_1_, and I_2_ are voltages and currents from cell_1_ and cell_2_, respectively, and *V_j_* = V_1_ − V_2_. To explore the influence of *V_j_* on *G_j_*, voltage steps of positive and negative polarities were applied only in cell_1_, and changes in holding currents were measured in cell_2_ while keeping V_2_ constant (I_j_ = −I_2_), and *G_j_* = I_j_/*V_j_*. Brief V_j_ pre-pulses of −20 mV were applied before each *V_j_* step to control the stability of *G_j_* between *V_j_* steps. *G_j_*–*V_j_* relationships were fitted using a two-state Boltzmann equation as previously described [26]:

(3) $$G_{j}=\frac{Gmax-Gmin}{1+\exp\left[A*\left(V_{j}-V_{0}\right)\right]}+Gmin$$

where *V_0_* represents the transjunctional voltage at which *Gj* is half-maximal, and constant *A* defines the steepness of *V_j_*-sensitivity. *Gmax* and *Gmin* represent maximum and minimum conductance, respectively. Recordings and data analysis were performed using P-clamp software (Axon Instruments, CA, USA) and Digidata 1322A (Axon Instruments). Measured currents were low-pass filtered at 2 kHz and sampled at a rate of 50–100 µs.

### 2.10. Statistical Analyses

Statistical analyses were computed using Prism 5 (GraphPad software, La Jolla, CA, USA). Student’s t-test or one-way ANOVA with post-hoc Dunnett’s multiple comparison was performed, and *p*-values < 0.05 were considered statistically significant.

## 3. Results

### 3.1. Functional Expression of Endogenous GJs in HeLa Cells

**Figure 1:**
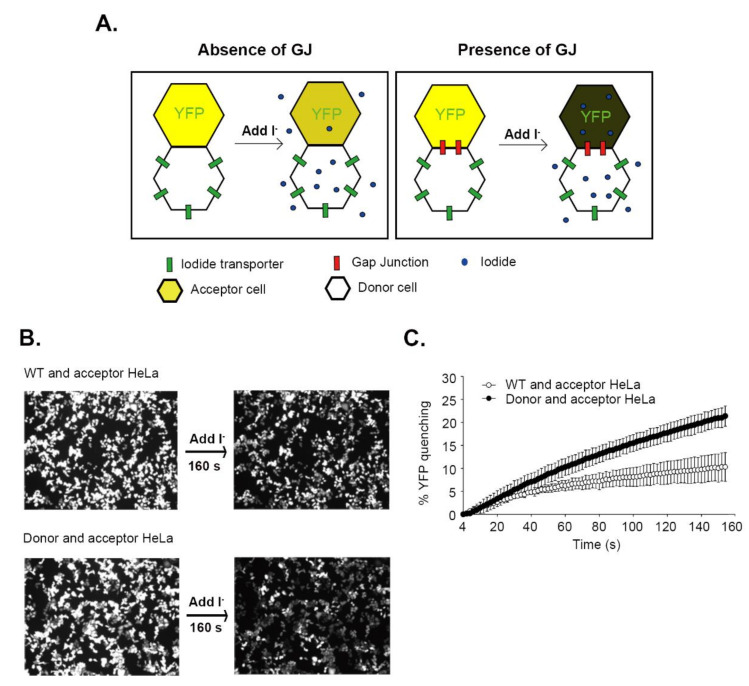
I-YFP assay revealed GJIC activity in HeLa cells. (**A**) A schematic presentation of the I-YFP GJIC assay. The yellow hexagon is an acceptor cell stably expressing YFP^QL^, a YFP variant whose fluorescence is quenched by iodides. The white hexagon with green rectangles is a donor cell that expresses SLC26A4, an iodide transporter. If donor and acceptor HeLa cells are connected by GJs (red rectangles), added iodides go through SLC26A4 and GJs and quench the YFP in the acceptor cell. If HeLa cells do not express functional GJs, iodides can enter the donor cell but cannot diffuse to acceptor cells. A single iodide in the acceptor cell of the left panel denotes non-specific and slow iodide influx into the HeLa cell. The degree of YFP quenching is presented as a dark yellow color in the acceptor cells. (**B**) Fluorescence images of the I-YFP GJIC assay in HeLa cells. The acceptor HeLa cells combined with WT (top) or donor (bottom) HeLa cells were plated on a six-well plate. After a 24-h incubation, the culture media were replaced with 1 mL of C-solution. The fluorescence images were taken before and 160 s after adding 1 mL I-solution on a fluorescence microscope (Observer.D1, Zeiss). (**C**) I-YFP GJIC assay in HeLa cells. WT and acceptor HeLa cells or donor and acceptor HeLa cells were mixed at a ratio of 4:1 and plated on 96-well plates before the I-YFP GJIC assay. The YFP quenching percentage was plotted against assay time. The data are expressed as the mean ± SD (*n* = 9).

We used the I-YFP GJIC assay to examine whether HeLa cells have GJ activity [18,27,28,29]. The principle of the assay is presented in Figure 1A. The assay utilizes a mixed culture of donor and acceptor cells that are engineered to stably express the iodide transporter SLC26A4 and YFP^QL^, respectively. Donor cells can rapidly take up iodides, while YFP fluorescence of acceptor cells can be sensitively reduced in the presence of intracellular iodides. If HeLa cells express functional GJs, the iodides enter donor cells via SLC26A4 when iodides are added to the co-culture of the donor and acceptor HeLa cells, diffuse to the acceptor cells through the GJs, and quench YFP^QL^ (Figure 1A, right box). If there are no functional GJs between the donor and acceptor HeLa cells, the iodides enter the donor cells without being transferred to acceptor cells or quenching YFP (Figure 1A, left box). The speed of the YFP quenching reflects GJIC activity. 

We generated donor and acceptor HeLa cells using lentiviral transduction. Acceptor HeLa cells mixed with wild type (WT) or donor HeLa cells were plated on a six-well plate at full confluence. After a 24-h incubation, the YFP fluorescence images were taken before and 160 s after I-solution injection. A greater YFP fluorescence reduction was observed in donor and acceptor HeLa cells than WT and acceptor HeLa cells (Figure 1B). We conducted the I-YFP GJIC assay with cells plated on 96-well plate and microplate reader to obtain more quantitative results. The YFP quenching value at 160 s was 10.75 ± 2.77% (*n* = 9) in the WT and acceptor group and 21.17 ± 2.18% (*n* = 9) in the donor and acceptor group (Figure 1C). The difference between the two groups demonstrated the GJIC activity endogenously expressed in HeLa cells. We also conducted scrape loading dye transfer assay but could not show LY diffusion via GJ (Figure S1), possibly due to weak GJIC in HeLa cells.

### 3.2. Identification of Cx45 as a Major Cx Isotype in HeLa Cells

**Figure 2:**
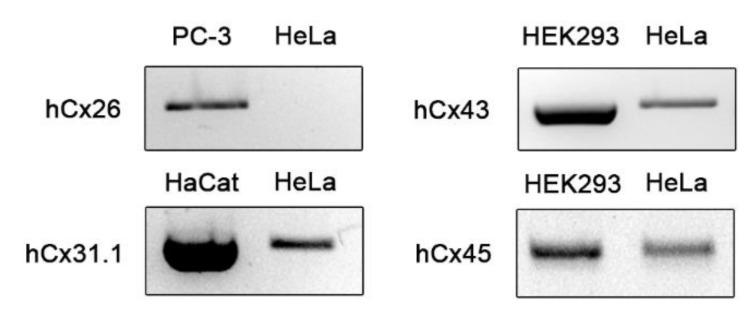
Cx expression at the mRNA level in HeLa cells. HeLa cell cDNA was amplified using PCR primer pairs specific for *GJB2* (Cx26), *GJB5* (Cx31.1), *GJA1* (Cx43), and *GJC1* (Cx45) shown in Table 2 and analyzed using 2% agarose gel electrophoresis. PC-3, HaCat, and HEK293 cell cDNAs were used as a positive control, as indicated above.

We performed RT-PCR analysis to identify Cx isotypes expressed in HeLa cells. The mRNA expression levels of *GJB2* (Cx26), *GJB5* (Cx31.1), *GJA1* (Cx43), and *GJC1* (Cx45) were investigated. We designed all RT-PCR primers (Table 2) to span two exons. The PCR result showed that, of the four members tested, *GJB5* (Cx31.1), *GJA1* (Cx43), and *GJC1* (Cx45) are expressed at the mRNA level in HeLa cells (Figure 2). Given that the *GJA1* (Cx43) PCR bands of HEK293 and HeLa cells seemed different in size, the PCR products were purified from the agarose gel and sequenced. The product from HEK293 was completely matched to *GJA1* (Cx43) (File S3). The sequence from HeLa cells was a mixture of *GJA1* (Cx43) and *GJA1P1*, a related pseudogene (File S4). To further validate this observation, we incubated the PCR products with ApoI which cleaves only *GJA1P1* products (see File S5 for the rationale of the ApoI discrimination). ApoI failed to digest the PCR product from HEK293 but digested about half of the PCR product from HeLa, which confirmed the expression of *GJA1P1* in HeLa cells (Figure S2).

**Figure 3:**
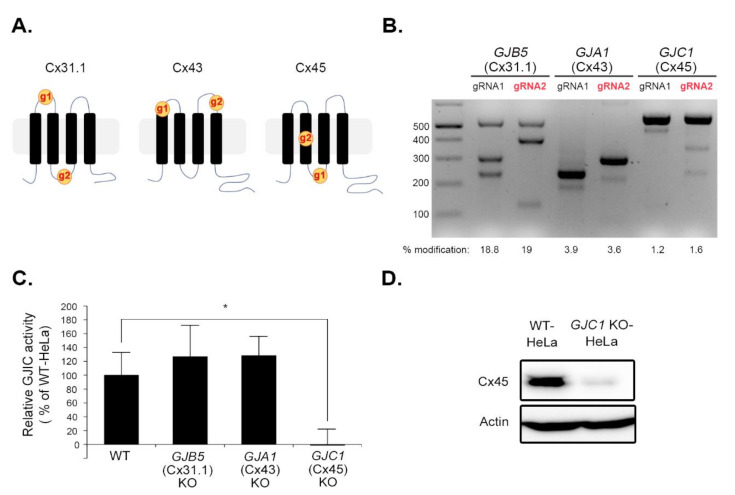
Genetic ablation of *GJC1* (Cx45) using the CRISPR/Cas9 system eliminated GJIC activity in HeLa cells. (**A**) The target positions of gRNAs used here were shown in the topological structures of Cx31.1, Cx43, and Cx45. (**B**) Selection of gRNAs for each Cx gene. HEK293T cells grown on a six-well plate were transfected with LentiCRISPRv2 plasmid bearing each gRNA and cultivated for 48 h. Transfected cells were selected with 2 μg/mL puromycin for 48 h. Genomic DNA was extracted from the surviving cells for the T7E1 assay. PCR was conducted with primer pairs shown in Table 3. PCR products were re-annealed, digested with T7E1, and analyzed using 2% agarose electrophoresis. The gene modification percentage was calculated using Equation (2) and presented below the gel image. The efficient gRNAs were selected (red) based on the modification percentage. (**C**) *GJB5* (Cx31.1) KO-, *GJA1* (Cx43) KO-, *GJC1* (Cx45) KO-HeLa cells were generated using transduction with the LentiCRISRv2 virus expressing each gRNA selected above. Next, donor and acceptor cells from each KO cell were obtained using transduction with pLVX-CIH-SLC26A4 and pLVX-CIBla-YFP^QL^ viruses, respectively. The I-YFP GJIC assay was conducted with each donor and acceptor pair. The GJIC activity was calculated as the percentage of WT HeLa cells and expressed as the mean ± SD of three independent experiments per group in the bar graph. * *p* < 0.05 vs. WT by t-test (*n* = 3). Each independent experiment started from the generation of a different batch of KO cells. (**D**) Whole lysates were prepared from WT and *GJC1* (Cx45) KO-HeLa cells grown on a six-well plate and analyzed using immunoblotting with anti-Cx45 and anti-actin antibodies.

We designed two or three gRNAs for each Cx gene and assessed their efficiency using a T7E1 assay to knock out *GJB5* (Cx31.1), *GJA1* (Cx43), and *GJC1* (Cx45) in HeLa cells with the CRISPR/Cas9 system. The locations of the gRNA targets were shown in the topological structure of each Cx gene (Figure 3A). The two gRNAs for *GJB5* (Cx31.1) or *GJC1* (Cx45) were assessed with identical PCR primers (Table 3) due to their vicinity. In the case of *GJA1* (Cx43), nested PCR was performed to minimize the contamination of *GJA1P1*, a pseudogene of *GJA1* [30]. Although the primers for the second (inner) PCR cannot discriminate between *GJA1* (Cx43) and *GJA1P1*, since the first (outer) PCR primers were selected to bind only to *GJA1* (Cx43) but not to *GJA1P1*, the nested PCR amplified only the *GJA1* (Cx43) locus. Based on the modification in Figure 3B, gRNA2s were chosen for *GJB5* (Cx31.1), *GJA1* (Cx43), and *GJC1* (Cx45). Off-target sites (≤2-base mismatch) for the selected gRNAs were found using Cas-OFFinder [31] and are presented in File S6. No off-target sites were found within the exons of protein-coding genes.

*GJB5* (Cx31.1) KO-, *GJA1* (Cx43) KO-, and *GJC1* (Cx45) KO-HeLa cells were prepared using transduction with LentiCRISPRv2 virus expressing spCas9 and each gRNA selected above. Next, donor and acceptor cells for the I-YFP GJIC assay were generated for each KO cell. The I-YFP GJIC assay showed a slight increase in GJIC activity following *GJB5* (Cx31.1) KO (125.94 ± 6.24%) and *GJA1* (Cx43) KO (132.94 ± 23.69%). However, most GJIC activity was lost in *GJC1* (Cx45) KO-HeLa cells (2.45 ± 22.24%) (Figure 3C). Since the Cx KO-induced changes in iodide permeability in acceptor cells might have led to these results, we also conducted the I-YFP GJIC assay with WT acceptor cells and KO donor cells. The results (Figure S3) show the same pattern (Figure 3C). The *GJC1* (Cx45) KO was confirmed at the protein level using immunoblot analysis (Figure 3D). The immunofluorescent staining failed to show GJ signal at the junctions of adjacent cells (Figure S4), which was similar to the result of a previous report [21] and might have been due to its weak expression. In addition, the mRNA expression levels of Cx26, Cx31.1, and Cx43 were not reduced by *GJC1* (Cx45) KO (Figure S5 and File S7).

### 3.3. Electrophysiological Measurement of GJ Activity in HeLa Cells

**Figure 4:**
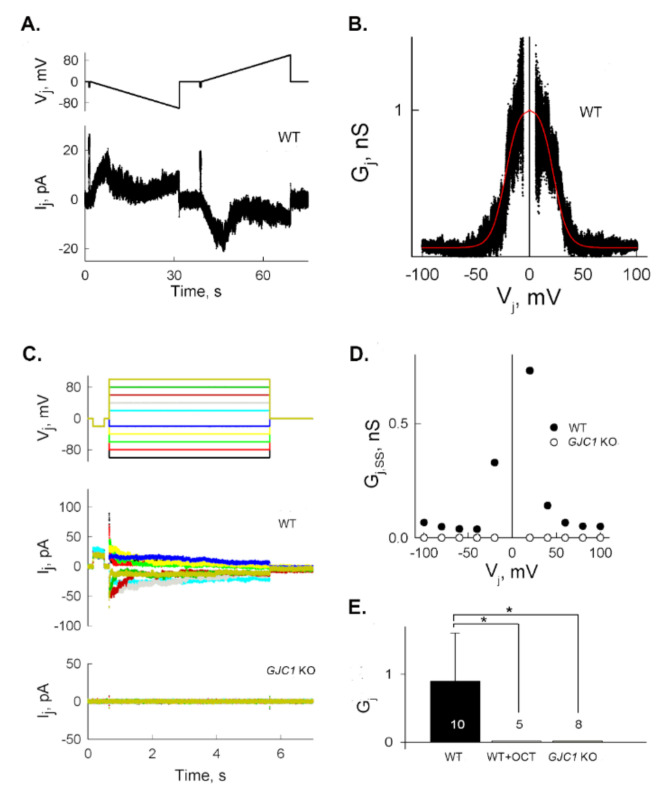
Electrical coupling and V_j_-gating are abolished by knocking out *GJC1* (Cx45) in HeLa cells. (**A**) Example of macroscopic transjunctional currents (I_j_, bottom trace) recorded in response to long transjunctional voltage ramps (~3.5 mV/s) from 0 to +100 and −100 mV (V_j_, top trace) in a WT HeLa cell pair. (**B**) Junctional conductance dependence over transjunctional voltage (G_j_–V_j_ plot), calculated from the record shown in (A). The red spline shows fitting with a two-state Boltzmann equation; parameters Vo = 21.8 mV and A = 0.153 mV^−1^. (**C**) Macroscopic transjunctional currents (I_j_) in response to stepwise V_j_ pulses from ±20 to ±100 mV, applied via 20-mV increments (top trace) to WT (middle trace) or *GJC1* (Cx45) KO-HeLa (bottom trace) cell pairs. (**D**) Steady-state junctional conductance (G_j,ss_) of WT (filled circles) and *GJC1* (Cx45) KO-HeLa (open circles) cell pairs at different V_j_, calculated from records shown in C. (**E**) Average macroscopic G_j_ measured at −20 mV in WT HeLa cells (WT), WT HeLa cells treated with 1 mM octanol (WT + OCT), and *GJC1* (Cx45) KO-HeLa cells (*GJC1* (Cx45) KO) are presented as bar graphs. Digits within each bar correspond to the number of independent experiments. Data are presented as the mean ± SEM. * *p* < 0.05 vs. WT HeLa using t-test.

The dual whole-cell patch clamp technique was employed to confirm endogenous GJ activity in HeLa cells and its dependence on Cx45 expression. Macroscopic junctional currents were recorded in HeLa cells (80–100% confluence) during long range slow voltage ramps (Figure 4A). The steep voltage dependence of junctional conductance (G_j_) was fitted with a two-state Boltzmann equation (Equation (3)) to obtain the characteristics of V_j_-sensitivity for symmetric gates in series (Figure 4B), which were similar to the parameters of Cx45-based GJs [32,33]. Macroscopic junctional currents (I_j_) recorded in WT and *GJC1* (Cx45) KO-HeLa cell pairs in response to stepwise V_j_ pulses and the resultant steady-state junctional conductance (G_j,ss_) are shown in Figure 4C,D, respectively. I_j_ was ablated in *GJC1* (Cx45) KO cells. G_j,ss_ at −20 mV in WT HeLa cells was lost after treating with 1 mM octanol, a well-known GJ inhibitor [34], or absent in *GJC1* (Cx45) KO cells (Figure 4E).

### 3.4. Identification of the Cx Responsible for GJIC in A549 Cells

**Figure 5:**
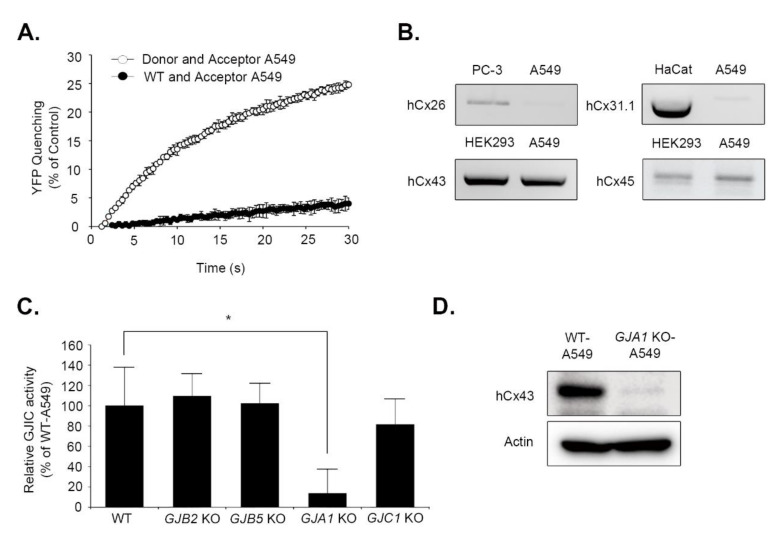
Identification of Cx43 as a major GJ component in A549 cells using the same experimental strategy as HeLa cells. (**A**) Acceptor A549 cells were mixed with WT or donor A549 cells at a ratio of 4:1 and plated on 96-well plates. The I-YFP GJIC assay was conducted after a 24 h-incubation. The YFP quenching percentage was plotted against assay time. The data are presented as the mean ± SD (*n* = 3). (**B**) A PCR was conducted with primers listed in Table 2 and A549, PC-3, HEK293, HaCat cDNA as templates as indicated above. The PCR products were analyzed using 2% agarose electrophoresis. (**C**) The GJIC activity in WT and each KO-A549 cells. The four Cx genes were knocked out using transduction with each LentiCRISPRv2 virus in A549 cells. Their donor and acceptor cells were generated using SLC26A4 and YFP^QL^ viruses, respectively, before the I-YFP GJIC assay. The GJIC activity percentage of WT A549 cells was calculated and presented as a bar graph. Data are presented as mean ± SD of three independent experiments per group. * *p* < 0.05 vs. WT A549 cells by t-test. Each independent experiment started from the generation of a different batch of KO cells. (**D**) Whole lysates of WT and *GJA1* (Cx43) KO-A549 cells were analyzed using immunoblotting with anti-Cx43 and anti-actin antibodies.

We conducted the I-YFP GJIC assay in A549 human lung cancer cells to examine whether our experimental strategy to identify the major Cx in cells with weak GJ activity could be applied to other cell types. The results show that the YFP quenching was higher in donor and acceptor A549 cells than in WT and acceptor A549 cells, with a difference of 21.14 ± 0.66% (*n* = 3) (Figure 5A). The mRNA expression of *GJB2* (Cx26), *GJB5* (Cx31.1)*, GJA1* (Cx43), and *GJC1* (Cx45) were detected, but their amounts varied (Figure 5B). Each of the four Cx genes was knocked out in A549 cells using the LentiCRISPRv2 virus. The gRNA for *GJB2* (Cx26) was selected based on the T7E1 assay result (Figure S6). Next, donor and acceptor cells were generated for each KO cell for the I-YFP GJIC assay. The KO of *GJB2* (Cx26), *GJB5* (Cx31.1), or *GJC1* (Cx45) did not attenuate GJIC in A549 cells, and GJ activity was reduced to 9.67 ± 16.66% due to the *GJA1* (Cx43) KO (Figure 5C). Immunoblot analysis (Figure 5D) and fluorescent immunostaining (Figure S7) confirmed *GJA1* (Cx43) KO at the protein level. The mRNA expression of Cx26, Cx31.1, and Cx45 were not reduced by *GJA1* (Cx43) KO (Figure S8 and File S7). Therefore, these results indicate that Cx43, but not Cx26, Cx31.1, or Cx45, plays a major role in GJIC in A549 cells 

## 4. Discussion

The mRNA expression of *GJB2* (Cx26)*, GJB5* (Cx31.1), and *GJC1* (Cx45) were reported in HeLa cells [35]. *GJA1* (Cx43) is the most ubiquitously expressed member of the Cx gene family [36]. Thus, we analyzed the four Cx genes via RT-PCR in HeLa cells and detected the mRNA expression of three of the Cx-coding genes, with the exception of *GJB2* (Cx26). The I-YFP GJIC assay following gene KO via the CRISPR/Cas9 system revealed Cx45 as the major Cx forming functional GJs in HeLa cells. We also used a dual whole-cell patch clamp to show that electrical coupling in WT HeLa cells depends on endogenous Cx45, since its electrophysiological properties and steep transjunctional voltage dependence were similar to those of previously reported Cx45-based GJs and disappears after knocking out *GJC1* (Cx45). The biophysical properties of endogenous GJs present in monolayer cultures of HeLa cells have been described to possess lower voltage sensitivity than that in our results (V_0_ of 49 mV vs. 22 mV) [20]. However, in both studies, HeLa cells showed a very weak GJIC. GJs formed of endogenous Cx45 were reported to have a V_0_ of 13.4 mV in SKHep1 cells [37] and 15 mV in rat Schwann cells [32], which are closer to those described in our results. Measurements of Cx45 single GJ channel conductance depends on species and range from 26 to 32 pS [38]. Sahu et al. [35] also showed a weak GJIC with steep voltage dependence, a characteristic of Cx45 in non-transfected HeLa cells. These previous views were corroborated here using the CRISPR/Cas9 system. The comparison of this study to the aforementioned reports suggests that Cx45-dependent GJ expression in HeLa cells, despite the heterogeneity of HeLa cells in genomics and transcriptomics across laboratories [39], might be a universal phenomenon. In general, HeLa cells can withstand whole-cell patch clamping for a sufficiently long time and are suitable for GJIC studies using exogenous Cx expression. *GJC1* (Cx45)-KO HeLa cells are more appropriate than WT HeLa cells as GJ-null cells for studies regarding the exogenous expression of different Cxs or pannexin channels.

We assessed the GJIC using the I-YFP assay, whose unique advantage is that higher sensitivity can be obtained using longer measurement times [18]. The I-YFP assay for 160 s per well detected a weak junctional coupling in HeLa cells that can be ignored in the experiments in transfected cells. While the dual whole-cell patch clamp technique measures junctional conductance in a quantitative manner, the I-YFP assay is semi-quantitative. Nonetheless, since the electrophysiological method is technically demanding, the I-YFP assay has a strong merit over the patch clamp method. The highly sensitive I-YFP GJIC assay can be easily established by transducing cells of interest with two lentivirus types. Additionally, knocking out each Cx gene make it possible to determine which Cx is crucial for the GJIC in the cells.

The experimental strategy in this study was successfully applied to A549 lung cancer cells, which are known to have a weak GJ activity [40,41]. The GJIC was stronger in A549 cells than in HeLa cells, and it was dependent on Cx43 expression. The application of this strategy to other cell types with GJIC activity can lead to the elucidation of major Cx types in the cells and the simultaneous establishment of a corresponding GJ assay system.

Cx45 may play an important role in propagating electrical activity in the mammalian heart [42] and regulating blood pressure [43]. Furthermore, Cx45 is present at some electrical synapses in the retina [44,45], and is associated with neural precursor cell proliferation [46] and migration [47]. However, there is much to be elucidated regarding the physiological roles of Cx45. The I-YFP GJ assay performed in HeLa cells can be optimized for use in the identification of Cx45-selective GJ modulators, which will help to study the functional roles of Cx45 in vivo. 

## 5. Conclusions

Our findings demonstrate that HeLa cells express GJs composed of Cx45 and can be used as a model cell for Cx45-related studies and that A549 cells express Cx43-based GJs. In addition, *GJC1* (Cx45)-KO HeLa cells are more appropriate than WT HeLa cells for use as GJ-null cells.

## Figures and Tables

**Table 1:** Oligonucleotides for LentiCRISPRv2 constructs.

sgRNA	Sequences
*GJB2* (Cx26) gRNA1	sense: 5′-CACCGCCTCCTTTGCAGCCACAACG-3′
	antisense: 5′-AAACCGTTGTGGCTGCAAAGGAGGC-3′
*GJB2* (Cx26) gRNA2	sense: 5′-CACCGTCCACGCCAGCGCTCCTAG-3′
	antisense: 5′-AAACCTAGGAGCGCTGGCGTGGAC-3′
*GJB5* (Cx31.1) gRNA1	sense: 5′-CACCGAACTCATCAAAGCAGACGT-3′
	antisense: 5′-AAACACGTCTGCTTTGATGAGTTC-3′
*GJB5* (Cx31.1) gRNA2	sense: 5′-CACCGGGCGCCTCTACCTGAACCC-3′
	antisense: 5′-AAACGGGTTCAGGTAGAGGCGCCC-3′
*GJA1* (Cx43) gRNA1	sense: 5′-CACCGAATCCTGCTGCTGGGGACA-3′
	antisense: 5′-AAACCTGTCCCCAGCAGCAGGATT-3′
*GJA1* (Cx43) gRNA2	sense: 5′-CACCGTTTTCTCCGTGGGGCGAGAG-3′
	antisense: 5′-AAACCTCTCGCCCCACGGAGAAAA-3′
*GJA1* (Cx43) gRNA3	sense: 5′-CACCGCACCACTGGTCGCATGGTAA-3′
	antisense: 5′-AAACTTACCATGCGACCAGTGGTG-3′
*GJC1* (Cx45) gRNA1	sense: 5′-CACCGCTAAGCATGATGGCCGACGA-3′
	antisense: 5′-AAACTCGTCGGCCATCATGCTTAGC-3′
*GJC1* (Cx45) gRNA2	sense: 5′-CACCGATAGCCCAGGTACATCACAG-3′
	antisense: 5′-AAACCTGTGATGTACCTGGGCTATC-3′

**Table 2:** Primers for RT-PCR.

Target	Sequences	Amplicon Size
*GJB2* (Cx26)	F: 5′-TTCCTCCCGACGCAGAGCAAAC-3′	428 bp
	R: 5′-AGCCTTCGATGCGGACCTTCTG-3′	
*GJB5* (Cx31.1)	F: 5′-GCTGCTTGCTGAGTCCTATTGCC-3′	514 bp
	R: 5′-TCCACGCTCGCCTTGAACACTAG-3′	
*GJA1* (Cx43)	F: 5′-AGGCGTGAGGAAAGTACCAAACAG-3′	461 bp
	R: 5′-CGCATCACATAGAACACATGAGCCAG-3′	
*GJC1* (Cx45)	F: 5′-AGGAGAGGCGAGGGTGAAGG-3′	545 bp
	R: 5′-TCCGAGCTGCCTTCTTGTCTGC-3′	

**Table 3:** PCR primers for T7E1 assay.

Target Locus	Sequences	Amplicon Size
*GJB2* (Cx26) gRNA1 & 2	F: TGCTTACCCAGACTCAGAGAAG	547 bp
	R: ATGACATAGAAGACGTACATGAAG	
*GJB5* (Cx31.1) gRNA1 & 2	F: TGTTCTTGTTTCCCTGCAGTAG	527 bp
	R: CCACAGGAGGGAGGATATATTTG	
*GJA1* (Cx43) gRNA1, 2, & 3 (outer PCR)	F: AGGGAAGGTGTGGCTGTCAGTAC	1200 bp
	R: ATAAGGCTGTTGAGTACCACC	
*GJA1* (Cx43) gRNA1 (inner PCR)	F: AGGGAAGGTGTGGCTGTCAGTAC	204 bp
	R: CGCATCACATAGAACACATGAGCCAG	
*GJA1* (Cx43) gRNA2 & 3 (inner PCR)	F: GTGGTACATCTATGGATTCAGCTTGAGTG	303 bp
	R: GGTGAGGAGCAGCCATTGAAATAAGC	
*GJC1* (Cx45) gRNA1 & 2	F: TAGAGGAGATTCACAACCATTC	590 bp
	R: AAACGGGTGGACTTGGAAG	

## Supplementary Materials

The following are available online at https://www.mdpi.com/2218-273X/10/10/1389/s1, Figure S1: Scrape loading dye transfer assay, Figure S2: Presence of *GJA1P1* in PCR product using GJA1 (Cx43) F and R primers, Figure S3: I-YFP GJIC assay using WT acceptor and KO donor cells, Figure S4: Immunofluorescence of WT-and *GJC1* (Cx45) KO-HeLa cells, Figure S5: mRNA expression of Cx26,Cx31.1, and Cx43 in WT- and *GJC1* (Cx45) KO-HeLa cells, Figure S6: Selection of gRNAs for *GJB2* (Cx26), Figure S7: Immunofluorescence of WT- and *GJA1* (Cx43) KO-A549 cells, Figure S8: mRNA expression of Cx26, Cx31.1, and Cx45 in WT- and *GJA1* (Cx43) KO-A549 cells, File S1: The cell line authentication of HeLa, *GJC1* KO-HeLa, A549, and *GJA1* KOA549 cells, File S2: Phase contrast images of HeLa, A549, and their derivatives, File S3: Sequencing result of Cx43 RT-PCR product from HEK293 cDNA, File S4: Sequencing result of Cx43 RT-PCR product from HeLa cDNA, File S5: Alignment of *GJA1* cDNA and *GJA1P1* that shows the residues that can discriminate the two sequences, File S6: Potential off-target loci for gRNAs used in this study, File S7: RT-PCR results at different PCR cycles.
