# Supplementary material for: Confirmation of Connexin45 Underlying Weak Gap Junctional Intercellular Coupling in HeLa Cells
Source: Biomolecules. 2020 Sep 29;10(10):1389. doi: 10.3390/biom10101389 (PMC7650549; doi:10.3390/biom10101389)

File S1. The cell line authentication of HeLa, *GJC1* KO-HeLa, A549, and *GJA1* KO-A549 cells. Korean Cell Line Bank analyzed the STR profile of the cells using STR kit described below.

STR Profile Results

Sample	D8S1179	D21S11	D7S820	CSF1PO	D3S1358	TH01	D13S317	D16S539
**HeLa (ATCC CCL-2)**	**12,13**	**27,28**	**8,12**	**9,10**	**15,18**	**7**	**12,14**	**9,10**
HeLa	12,13	27,28	8,12	9,10	15,18	7	12,14	9,10
HeLa-Cx45KO	12,13	27,28	8,12	9,10	15,18	7	12,14	9,10
**A549 (ATCC CCL-185)**	**13,14**	**29**	**8,11**	**10,12**	**16**	**8,9.3**	**11**	**11,12**
A549	13,14	29	8,11	10,12	16	8,9.3	11	11,12
A549-Cx43KO	13,14	29	8,11	10,12	16	8,9.3	11	11,12

Sample	D2S1338	D19S433	Vwa	TPOX	D18S51	Amelogenin	D5S818	FGA
**HeLa (ATCC CCL-2)**	**17**	**13,14**	**16,18**	**8,12**	**16**	**X**	**11,12**	**18,21**
HeLa	17	13,14	16,18	8,12	16	X	11,12	18,21
HeLa-Cx45KO	17	13,14	16,18	8,12	16	X	11,12	18,21
**A549 (ATCC CCL-185)**	**24**	**13**	**14**	**8,11**	**14,17**	**X,Y**	**11**	**23**
A549	24	13	14	8,11	14,17	X,Y	11	23
A549-Cx43KO	24	13	14	8,11	14,17	X,Y	11	23

STR Kit : AmplFLSTR identifiler PCR Amplification kit (Applied Biosystems, Foster, CA, cat.4322288)

Analysis Methods **: 3530xL DNA Analyzer (Applied Biosystems, Foster, CA)**

: GeneMapper v5 (Applied Biosystems, Foster, CA)

The Fingerprinting of cell lines by 'AmplFLSTR identifiler PCR Amplification kit' was tested on July 29, 2020.

Korean Cell Line Bank
Cancer Research Institute
Seoul National University College of Medicine
Seoul, Korea
http://cellbank.snu.ac.kr
Tel : 82-2-3668-7915, Fax : 82-2-742-0021

Ja-Lok Ku, D.V.M, Ph.D.
Curator
Korean Cell Line Bank
E-mail :kujalok@snu.ac.kr

HeLa_STR results 2020.07.29

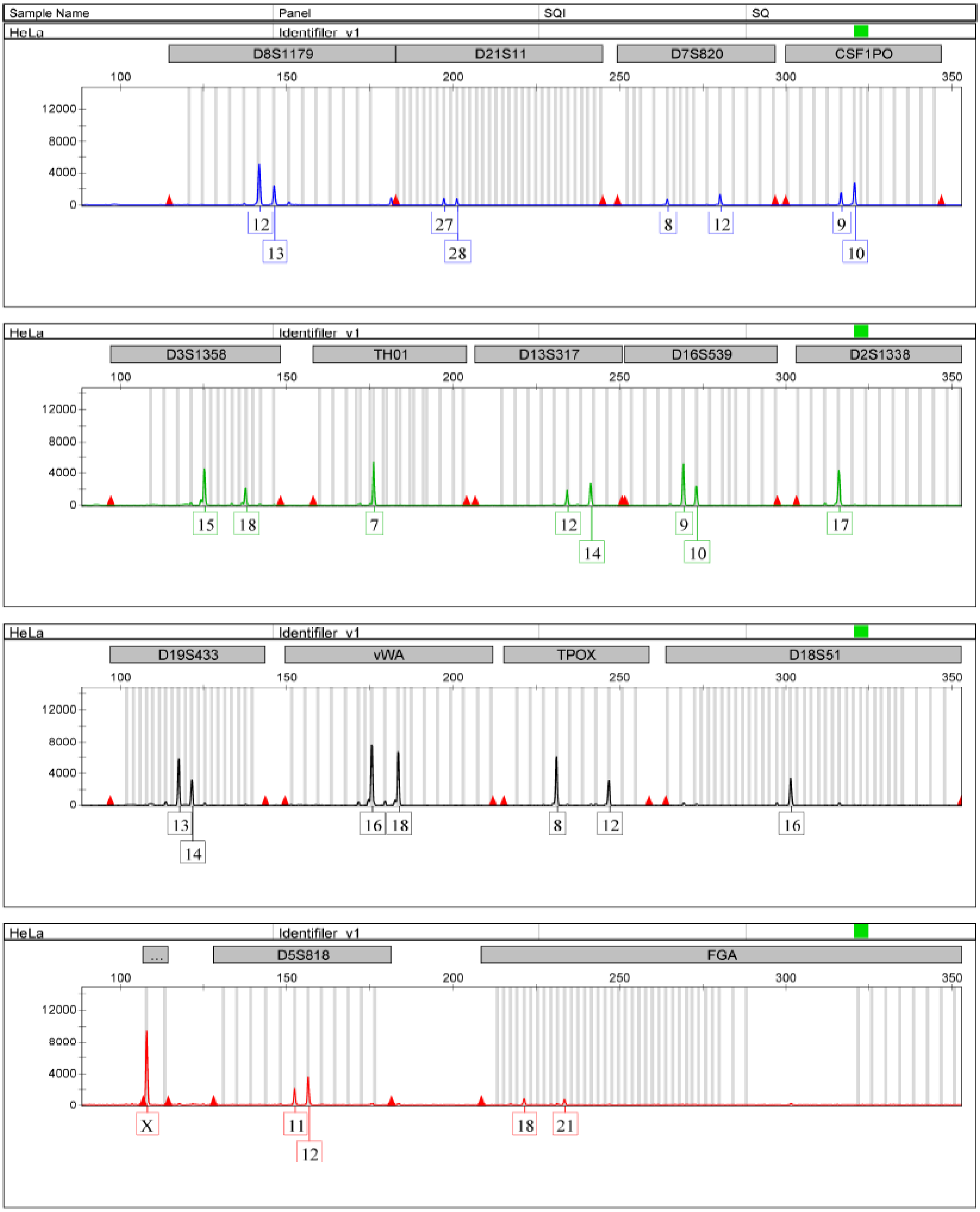

HeLa-Cx45KO_STR results 2020.07.29

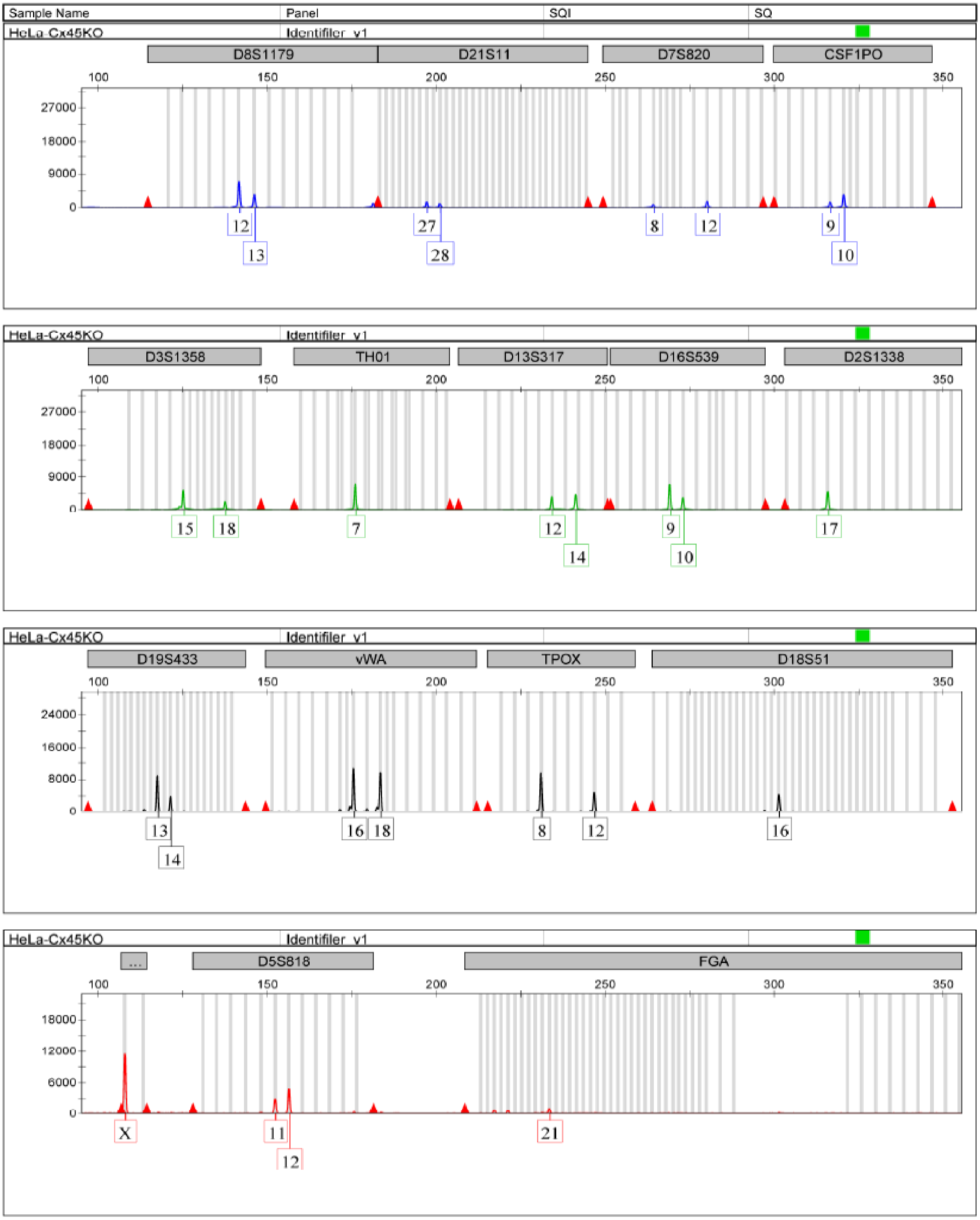

A549_STR results 2020.07.29

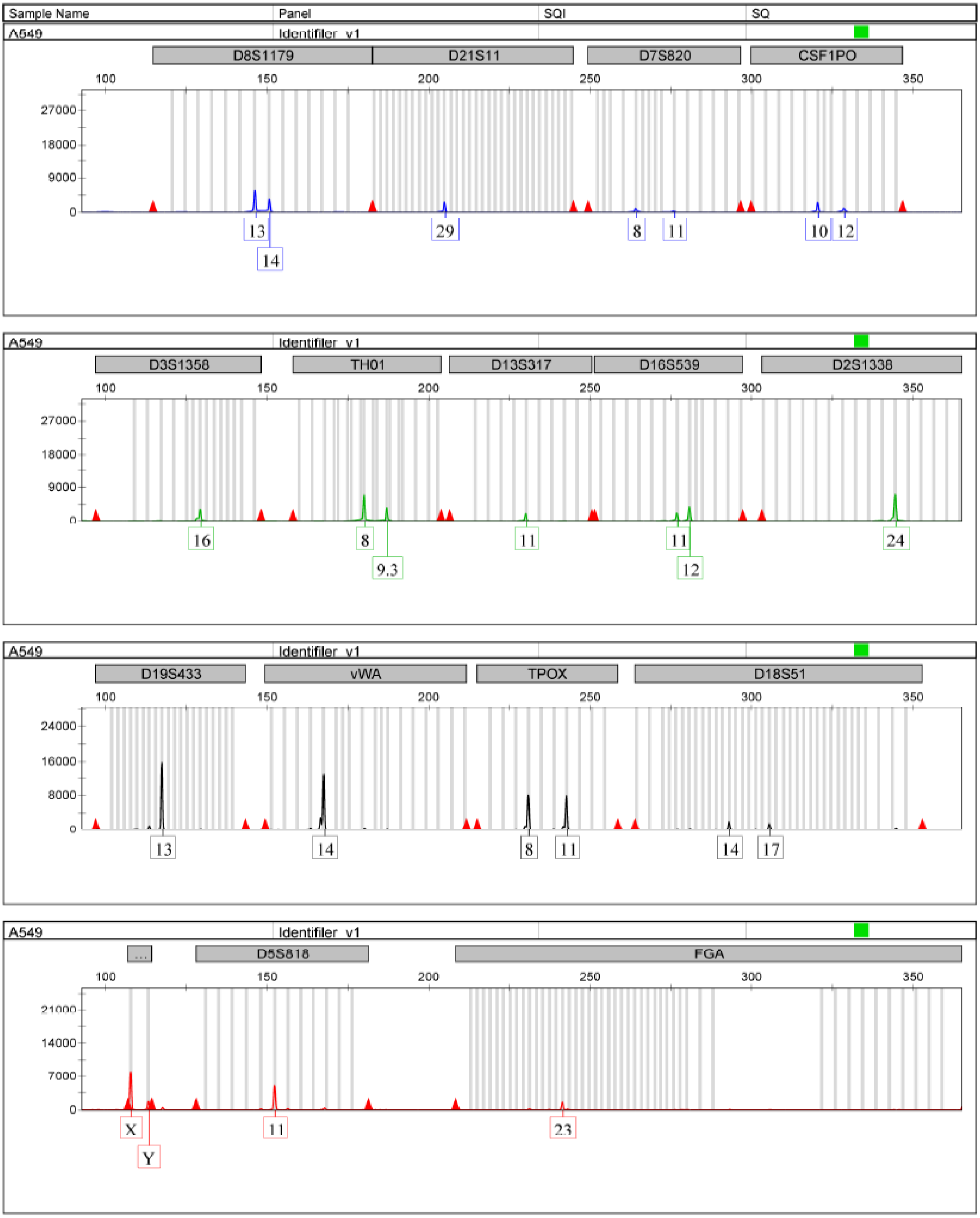

Korean Cell Line Bank

A549_Cx43KO_STR results 2020.07.29

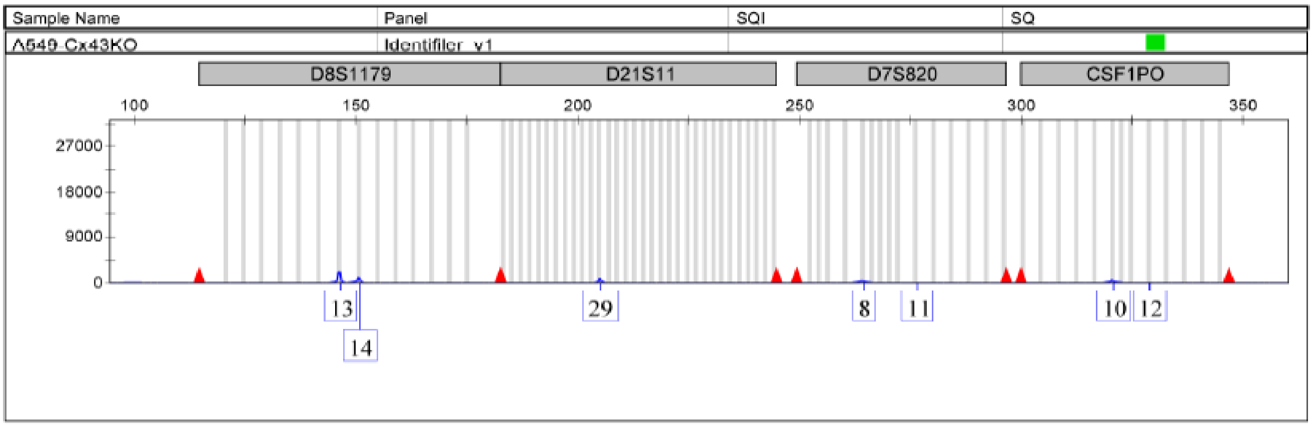

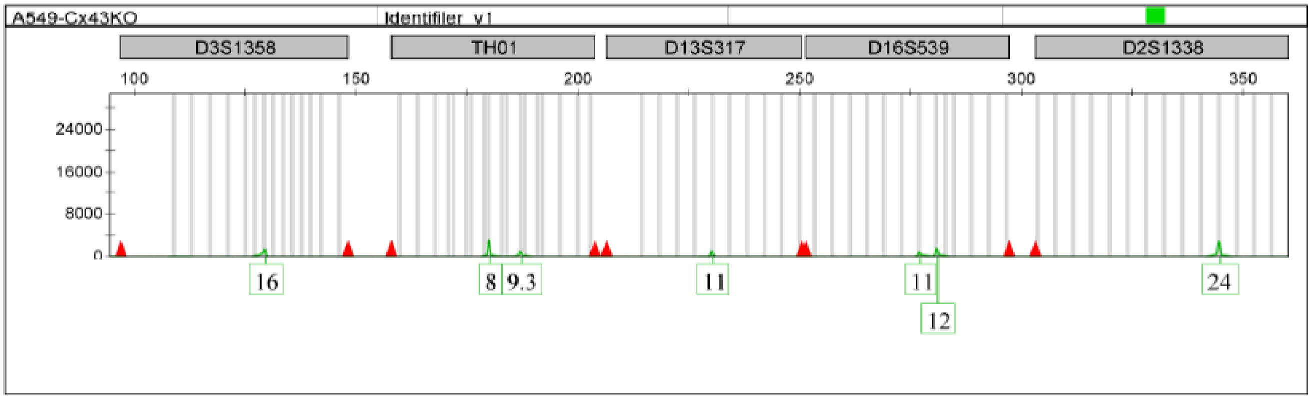

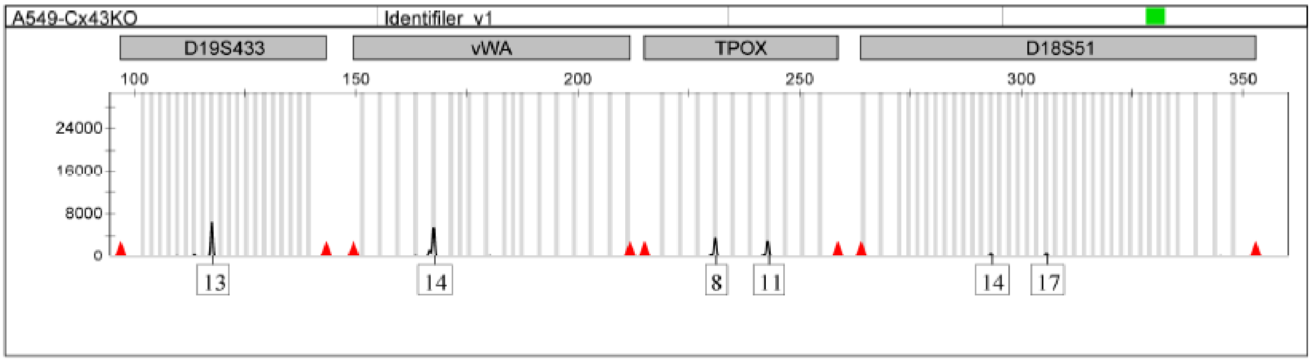

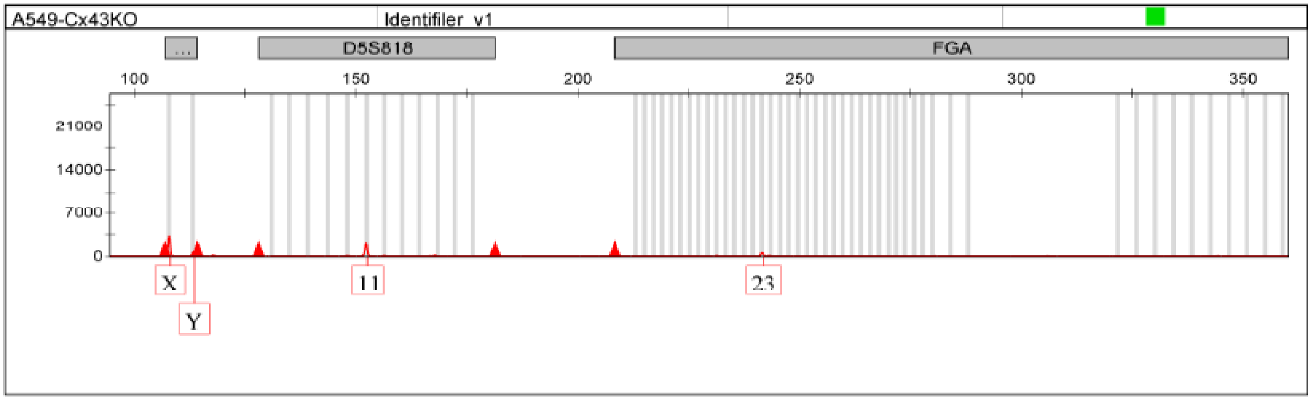

Supplement: Supplementary file 1 [file biomolecules-10-01389-s001.zip › File S.zip!/File S1.pdf]
