# Supplementary material for: Confirmation of Connexin45 Underlying Weak Gap Junctional Intercellular Coupling in HeLa Cells
Source: Biomolecules. 2020 Sep 29;10(10):1389. doi: 10.3390/biom10101389 (PMC7650549; doi:10.3390/biom10101389)

File S2. Phase contrast images of HeLa, A549, and their derivatives.

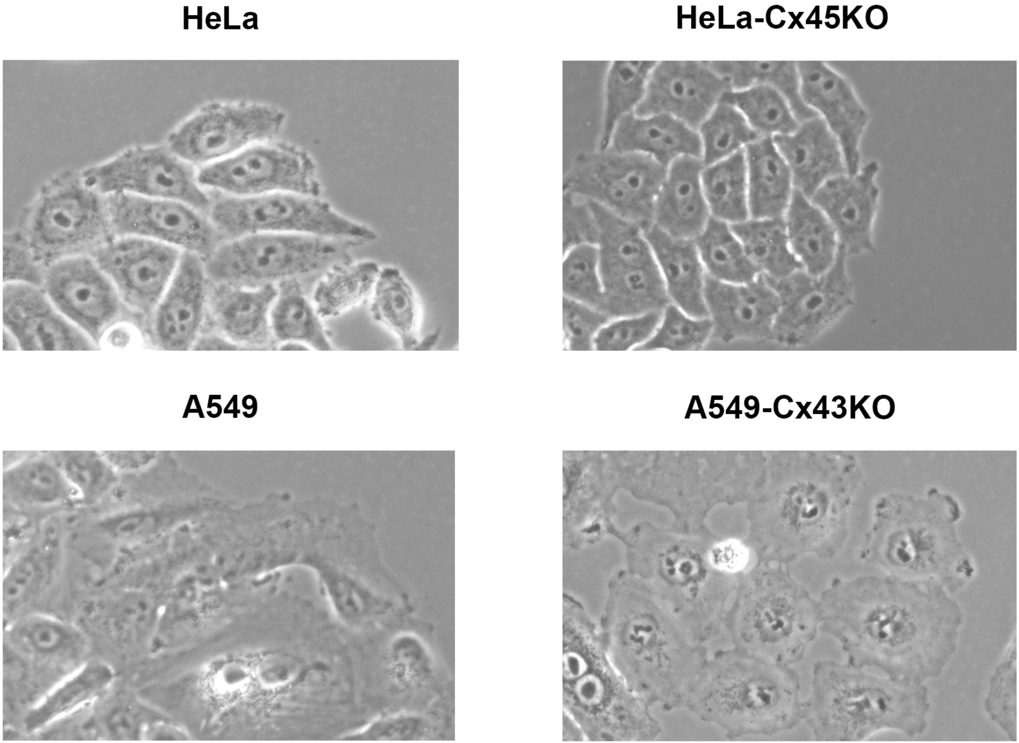

Supplement: Supplementary file 1 [file biomolecules-10-01389-s001.zip › File S.zip!/File S2.pdf]
