# Supplementary material for: Confirmation of Connexin45 Underlying Weak Gap Junctional Intercellular Coupling in HeLa Cells
Source: Biomolecules. 2020 Sep 29;10(10):1389. doi: 10.3390/biom10101389 (PMC7650549; doi:10.3390/biom10101389)
Supplement: Supplementary file 1 [file biomolecules-10-01389-s001.zip › File S.zip!/File S3.pdf]

File S3. Sequencing result of Cx43 RT-PCR product from HEK293 cDNA. Cyan rectangle denotes one of the residues that discriminate *GJA1* and *GJA1P1*. Only C (blue line) peak from *GJA1* was detected.

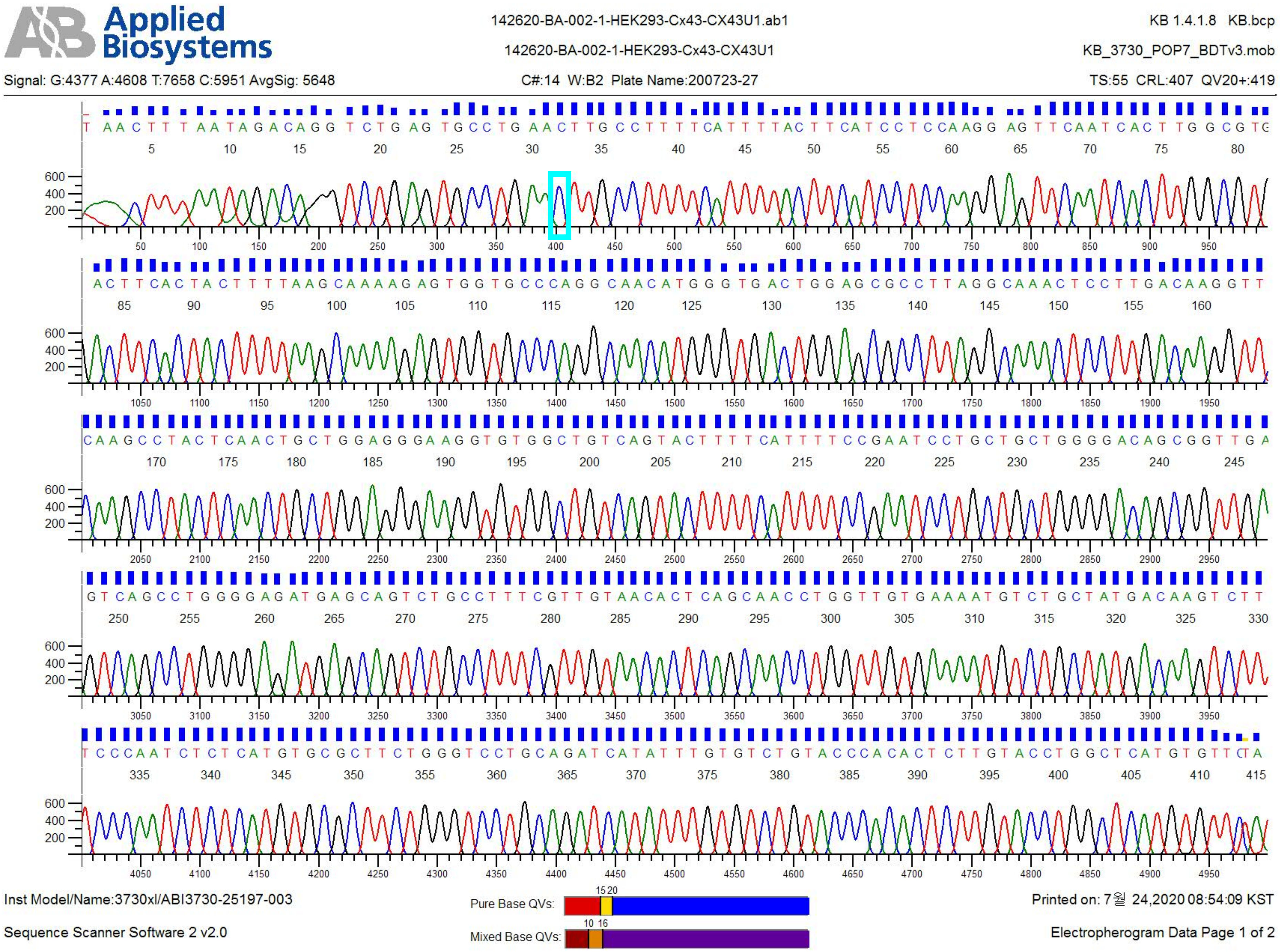

Signal: G:4377 A:4608 T:7658 C:5951 AvgSig: 5648

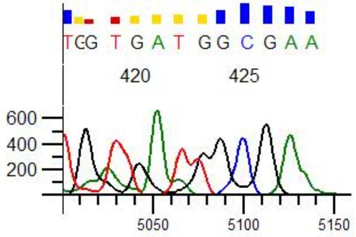

Inst Model/Name:3730xl/ABI3730-25197-003

Sequence Scanner Software 2 v2.0

Pure Base QVs: 15 20

Mixed Base QVs: 10 16

Printed on: 7월 24,2020 08:54:09 KST
