# Supplementary material for: Confirmation of Connexin45 Underlying Weak Gap Junctional Intercellular Coupling in HeLa Cells
Source: Biomolecules. 2020 Sep 29;10(10):1389. doi: 10.3390/biom10101389 (PMC7650549; doi:10.3390/biom10101389)

File S4. Sequencing result of Cx43 RT-PCR product from HeLa cDNA. Cyan rectangle denotes one of the residues that discriminate *GJA1* and *GJA1P1*. Mixed signal of C (blue line) from *GJA1* and T (red line) from *GJA1P1* was shown in the rectangle.

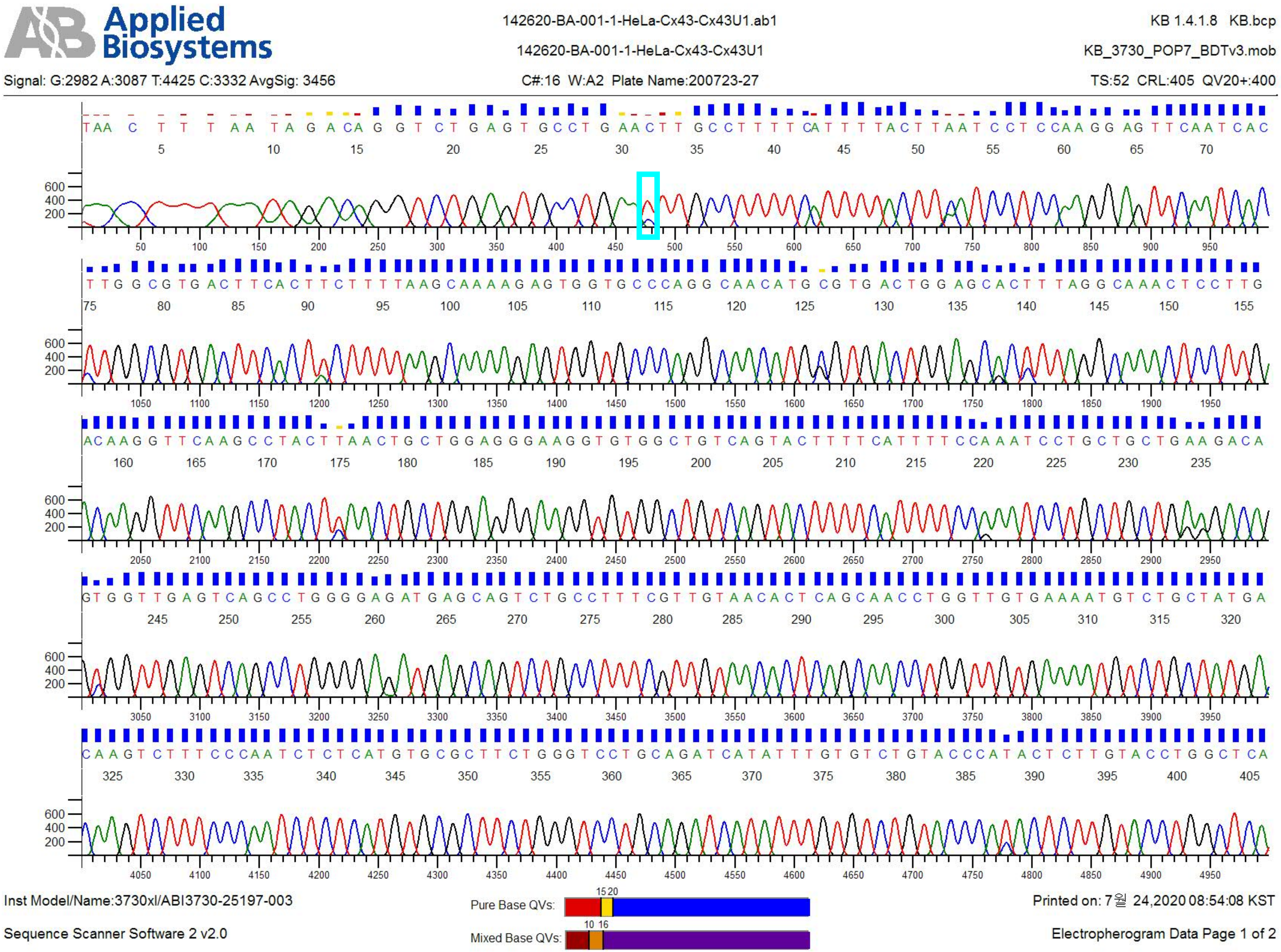

Signal: G:2982 A:3087 T:4425 C:3332 AvgSig: 3456

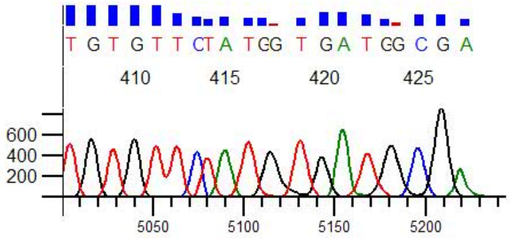

Supplement: Supplementary file 1 [file biomolecules-10-01389-s001.zip › File S.zip!/File S4.pdf]
