# Supplementary material for: Confirmation of Connexin45 Underlying Weak Gap Junctional Intercellular Coupling in HeLa Cells
Source: Biomolecules. 2020 Sep 29;10(10):1389. doi: 10.3390/biom10101389 (PMC7650549; doi:10.3390/biom10101389)
Supplement: Supplementary file 1 [file biomolecules-10-01389-s001.zip › File S.zip!/File S5.pdf]

File S5. Alignment of *GJA1* cDNA and *GJA1P1* that shows the residues that can discriminate the two sequences. The residues without vertical line are mismatches between them. Despite three mismatches in the PCR primer pair, *GJA1P1* was amplified by the primers (File S4). The amplicon only from *GJA1P1* can be digested by ApoI.

Accession No. of *GJA1* and *GJA1P1* are NM_000165.5 and NG_003029.5, respectively.
Yellow, *GJA1* F and R primer binding sites; Gray, residues that mismatch PCR primers; Cyan, ApoI site

```
GJA1   1      AAAAGCTTTTACGAGGTATCAGCACTTTTCTTTCATTAGGGGGAAGGCGTGAGGAAAGTA  60
              |   ||||||||||||||||||||||||||||||||| ||||||||| ||||||||||||
GJA1P1 1      A---GCTTTTACGAGGTATCAGCACTTTTCTTTCATT-GGGGGAAGGTGTGAGGAAAGTA  56

GJA1   61     CCAAACAGCAGCGGAGTTTTAAACTTTAAATAGACAGGTCTGAGTGCCTGAACTTGCCTT  120
               ||||||||||||||||||||||||||||||||||||||||||||||||||| |||||||
GJA1P1 57     ACAAACAGCAGCGGAGTTTTAAACTTTAAATAGACAGGTCTGAGTGCCTGAATTTGCCTT  116

GJA1   121    TTCATTTTACTTCATCCTCCAAGGAGTTCAATCACTTGGCGTGACTTCACTACTTTTAAG  180
              ||||||||||||||||||||||||||||||||||| ||||||||||||||| ||||||||
GJA1P1 117    TTCATTTTACTTCATCCTCCAAGGAGTTCAATCACCTGGCGTGACTTCACTTCTTTTAAG  176

GJA1   181    CAAAAGAGTGGTGCCCAGGCAACATGGGTGACTGGAGCGCCTTAGGCAAACTCCTTGACA  240
              |||||||||||||||||||||||||| ||||||||||| | |||||||||||||||||||
GJA1P1 177    CAAAAGAGTGGTGCCCAGGCAACATGCGTGACTGGAGCACTTTAGGCAAACTCCTTGACA  236

GJA1   241    AGGTTCAAGCCTACTCAACTGCTGGAGGGAAGGTGTGGCTGTCAGTACTTTTCATTTTCC  300
              ||||||||||||||| ||||||||||||||||||||||||||||||||||||||||||||
GJA1P1 237    AGGTTCAAGCCTACTTAACTGCTGGAGGGAAGGTGTGGCTGTCAGTACTTTTCATTTTCC  296

GJA1   301    GAATCCTGCTGCTGGGGACAGCGGTTGAGTCAGCCTGGGGAGATGAGCAGTCTGCCTTTC  360
               |||||||||||||  ||||| ||||||||||||||||||||||||||||||||||||||
GJA1P1 297    AAATCCTGCTGCTGAAGACAGTGGTTGAGTCAGCCTGGGGAGATGAGCAGTCTGCCTTTC  356

GJA1   361    GTTGTAACACTCAGCAACCTGGTTGTGAAAATGTCTGCTATGACAAGTCTTTCCCAATCT  420
              ||||||||||||||||||||||||||||||||||||||||||||||||||||||||||||
GJA1P1 357    GTTGTAACACTCAGCAACCTGGTTGTGAAAATGTCTGCTATGACAAGTCTTTCCCAATCT  416

GJA1   421    CTCATGTGCGCTTCTGGGTCCTGCAGATCATATTTGTGTCTGTACCCACACTCTTGTACC  480
              |||||||||||||||||||||||||||||||||||||||||||||||| |||||||||||
GJA1P1 417    CTCATGTGCGCTTCTGGGTCCTGCAGATCATATTTGTGTCTGTACCCATACTCTTGTACC  476

GJA1   481    TGGCTCATGTGTTCTATGTGATGCGAAAGGAAGAGAAACTGAACAAGAAAGAGGAAGAAC  540
              |||||||||||||||||||||||| |||||||||||||||||||||||||||||| ||||
GJA1P1 477    TGGCTCATGTGTTCTATGTGATGCAAAAGGAAGAGAAACTGAACAAGAAAGAGGAGGAAC  536

GJA1   541    TCAAGGTTGCCCAAACTGATGGTGTCAATGTGGACATGCACTTGAAGCAGATTGAGATAA  600
              |||||||||||||||||||||||||||||||||| |||||||||||||||||||||||||
GJA1P1 537    TCAAGGTTGCCCAAACTGATGGTGTCAATGTGGAGATGCACTTGAAGCAGATTGAGATAA  596

GJA1   601    AGAAGTTCAAGTACGGTATTGAAGAGCATGGTAAGGTGAAAATGCGAGGGGGGTTGCTGC  660
              ||||||||||||| ||||||||||||||||||||||||||||||| ||||||||||||||
GJA1P1 597    AGAAGTTCAAGTATGGTATTGAAGAGCATGGTAAGGTGAAAATGCAAGGGGGGTTGCTGC  656

GJA1   661    GAACCTACATCATCAGTATCCTCTTCAAGTCTATCTTTGAGGTGGCCTTCTTGCTGATCC  720
              |||||||||||||||||||||||||||||||||||||||||||| ||||   ||||||||
GJA1P1 657    GAACCTACATCATCAGTATCCTCTTCAAGTCTATCTTTGAGGTGACCTT---GCTGATCC  713

GJA1   721    AGTGGTACATCTATGGATTCAGCTTGAGTGCTGTTTACACTTGCAAAAGAGATCCCTGCC  780
              ||||||||||||||||||||||||||||||||||| ||||||||||||||||||||||||
GJA1P1 714    AGTGGTACATCTATGGATTCAGCTTGAGTGCTGTTCACACTTGCAAAAGAGATCCCTGCC  773

GJA1   781    CACATCAGGTGGACTGTTTCCTCTCTCGCCCCACGGAGAAAACCATCTTCATCATCTTCA  840
              |||||||||||||||| ||||||||| |||||| ||||||||||||||||||||||||||
GJA1P1 774    CACATCAGGTGGACTGCTTCCTCTCTTGCCCCATGGAGAAAACCATCTTCATCATCTTCA  833

GJA1   841    TGCTGGTGGTGTCCTTGGTGTCCCTGGCCTTGAATATCATTGAACTCTTCTATGTTTTCT  900
              |||||||||||||||||||||||||||||||||||||||||||||||||||| |||||||
GJA1P1 834    TGCTGGTGGTGTCCTTGGTGTCCCTGGCCTTGAATATCATTGAACTCTTCTACGTTTTCT  893
```

```
GJA1   901   TCAAGGGCGTTAAGGATCGGGTTAAGGGAAAGAGCGACCCTTACCATGCGACCAGTGGTG  960
             ||||||||||||||||| |||||||||||||||||||||||||||||||||||| |||
GJA1P1 894   TCAAGGGCGTTAAGGATTGGGTTAAGGGAAAGAGCGACCCTTACCATGCGACCACTGGCC  953

GJA1   961   CGCTGAGCCCTGCCAAAGACTGTGGGTCTCAAAAATATGCTTATTTCAATGGCTGCTCCT  1020
             |||||||||| |||||||||||||||||||||||||||||||||||||||||||||||||
GJA1P1 954   CGCTGAGCCCCGCCAAAGACTGTGGGTCTCAAAAATATGCTTATTTCAATGGCTGCTCCT  1013

GJA1   1021  CACCAACCGCTCCCCTCTCGCCTATGTCTCCTCCTGGGTACAAGCTGGTTACTGGCGACA  1080
             ||||||| |||||||||||||||||||||| |||||||||||||||||||| ||||||||
GJA1P1 1014  CACCAACTGCTCCCCTCTCGCCTATGTCTCTTCCTGGGTACAAGCTGGTTAATGGCGACA  1073

GJA1   1081  GAAACAATTCTTCTTGCCGCAATTACAACAAGCAAGCAAGTGAGCAAAACTGGGCTAATT  1140
             |||||||||||||||||||||||||| |||||||||||||||||||||||||||||||||
GJA1P1 1074  GAAACAATTCTTCTTGCCGCAATTACGACAAGCAAGCAAGTGAGCAAAACTGGGCTAATT  1133

GJA1   1141  ACAGTGCAGAACAAAATCGAATGGGGCAGGCGGGAAGCACCATCTCTAACTCCCATGCAC  1200
             ||||||||||||||||||||||||||||||||||||||||||||||||||||||| ||||
GJA1P1 1134  ACAGTGCAGAACAAAATCGAATGGGGCAGGCGGGAAGCACCATCTCTAACTCCCACGCAC  1193

GJA1   1201  AGCCTTTTGATTTCCCCGATGATAACCAGAATTCTAAAAAACTAGCTGCTGGACATGAAT  1260
             |||||||||||||||||||||||||||||||||||||||||||||||||||||||||||
GJA1P1 1194  AGCCTTTTGATTTCCCCGATGATAACCAGAATTCTAAAAAACTAGCTGCTGGACATGAAC  1253

GJA1   1261  TACAGCCACTAGCCATTGTGGACCAGCGACCTTCAAGCAGAGCCAGCAGTCGTGCCAGCA  1320
             ||||||||||||||||||||||||||| ||||||||||||||||||||||||||||||||
GJA1P1 1254  TACAGCCACTAGCCATTGTGGACCAGCAACCTTCAAGCAGAGCCAGCAGTCGTGCCAGCA  1313

GJA1   1321  GCAGACCTCGGCCTGATGACCTGGAGATCTAGATACAGGCTTGAAAGCATCAAGATTCCA  1380
             |||| |||| |||||||||||||||||||||||||||||||||||||| |||||||||||
GJA1P1 1314  GCAGGCCTCAGCCTGATGACCTGGAGATCTAGATACAGGCTTGAAAGTATCAAGATTCCT  1373

GJA1   1381  CTCAATTGTGGAGAAGAAAAAAGGTGCTGTAGAAAGTGCACCAGGTGTTAATTTTGATCC  1440
             |||| |||||||||||||||||||||||||||||||||||||||||||||||||||||||
GJA1P1 1374  CTCACTTGTGGAGAAGAAAAAAGGTGCTGTAGAAAGTGCACCAGGTGTTAATTTTGATCC  1433

GJA1   1441  GGTGGAGGTGGTACTCAACAGCCTTATTCATGAGGCTTAGAAAACACAAAGACATTAGAA  1500
             ||||||||||||| ||||||| ||||||||||||||||||||||||||||||||||||||
GJA1P1 1434  GGTGGAGGTGGTATTCAACAGTCTTATTCATGAGGCTTAGAAAACACAAAGACATTAGAA  1493

GJA1   1501  TACCTAGGTTCACTGGGGGTGTATGGGGTAGATGGGTGGAGAGGGAGGGGATAAGAGAGG  1560
             ||||||||||||||||||||||||||||||||||||||||||||||||||||||||||||
GJA1P1 1494  TACCTAGGTTCACTGGGGGTGTATGGGGTAGATGGGTGGAGAGGGAGGGGATAAGAGAGG  1553

GJA1   1561  TGCATGTTGGTATTTAAAGTAGTGGATTCAAAGAACTTAGATTATAAATAAGAGTTCCAT  1620
             ||||| ||||||||||||||||||||||||||| ||||||||||||||||||||||||||
GJA1P1 1554  TGCATATTGGTATTTAAAGTAGTGGATTCAAAAAACTTAGATTATAAATAAGAGTTCCAT  1613

GJA1   1621  TAGGTGATACATAGATAAGGGCTTTTTCTCCCCGCAAACACCCCTAAGAATGGTTCTGTG  1680
             ||  |||||||||||| ||||||||||||||| ||||||||||||||||||||||||| |
GJA1P1 1614  TACATGATACATAGATTAGGGCTTTTTCTCCCAGCAAACACCCCTAAGAATGGTTCTGGG  1673

GJA1   1681  TATGTGAATGAGCGGGTGGTAATTGTGGCTAAATATTTTTGTTTTACCAAGAAACTGAAA  1740
             |||||||||||| |||||||||||||||||||||||||||||||||||||||||||||||
GJA1P1 1674  TATGTGAATGAGTGGGTGGTAATTGTGGCTAAATATTTTTGTTTTACCAAGAAACTGAAA  1733

GJA1   1741  TAATTCTGGCCAGGAATAAATACTTCCTGAACATCTTAGGTCTTTTCAACAAGAAAAAGA  1800
             ||||||||||||||||||||||||||||||||||||||||||||||||||||||||||||
GJA1P1 1734  TAATTCTGGCCAGGAATAAATACTTCCTGAACATCTTAGGTCTTTTCAACAAGAAAAAGA  1793

GJA1   1801  CAGAGGATTGTCCTTAAGTCCCTGCTAAAACATTCCATTGTTAAAATTTGCACTTTGAAG  1860
             ||||||||||||||||||||||||||||||||||||||||||||||||||||||||||||
GJA1P1 1794  CAGAGGATTGTCCTTAAGTCCCTGCTAAAACATTCCATTGTTAAAATTTGCACTTTGAAG  1853

GJA1   1861  GTAAGCTTTCTAGGCCTGACCCTCCAGGTGTCAATGGACTTGTGCTACTATATTTTTTTA  1920
             || |||||||||||||||||||||||||||||||||||||||||||||||||||||||||
GJA1P1 1854  GTTAGCTTTCTAGGCCTGACCCTCCAGGTGTCAATGGACTTGTGCTACTATATTTTTTTA  1913
```

```
GJA1   1921  TTCTTGGTATCAGTTTAAAATTCAGACAAGGCCCACAGAATAAGATTTTCCATGCATTTG  1980
             ||||||||||||||||||||||||||||||||||||||||||||||||||||||||||||
GJA1P1 1914  TTCTTGGTATCAGTTTAAAATTCAGACAAGGCCCACAGAATAAGATTTTCCATGCATTTG  1973

GJA1   1981  CAAAT-----------ACGTATATTCTTTTTCCATCCACTTGCACAATATCATTACCATC  2029
              ||||           || ||||||  |||||||||||||||||| ||||||||||||||
GJA1P1 1974  AAAATCTCAGCATTATACATATATT--TTTTCCATCCACTTGCACCATATCATTACCATC  2031

GJA1   2030  ACTTTTTCATCATTCCTCAGCTACTACTCACATTCATTTAATGGTTTCTGTAAACATTTT  2089
             ||||||||||||||||||||||||||||||||||||||||||||||||||||||||||||
GJA1P1 2032  ACTTTTTCATCATTCCTCAGCTACTACTCACATTCATTTAATGGTTTCTGTAAACATTTT  2091

GJA1   2090  TAAGACAGTTGGGATGTCACTTAACATTTTTTTT---TTGAGCTAAAGTCAGGGAATCAA  2146
             ||||||||||||||||||||||||||||||||||   |||||||| ||||||||||||||
GJA1P1 2092  TAAGACAGTTGGGATGTCACTTAACATTTTTTTTCTTTTGAGCTAGAGTCAGGGAATCAA  2151

GJA1   2147  GCCATGCTTAATATTTAACAATCACTTATATGTGTGTCGAAGAGTTTGTTTTGTTTGTCA  2206
             |||||||||||||||||||| |||||| ||||||||| ||||  ||||||||||||||||
GJA1P1 2152  GCCATGCTTAATATTTAACAGTCACTTGTATGTGTGTGGAAG--TTTGTTTTGTTTGTCA  2209

GJA1   2207  TGTATTGGTACAAGCAGATACAGTATAAACTCACAAACACAGATTTGAAAATAATGCACA  2266
             ||||||||||||||||||||||||| ||||||||||||||||||||||||||||||||||
GJA1P1 2210  TGTATTGGTACAAGCAGATACAGTACAAACTCACAAACACAGATTTGAAAATAATGCACA  2269

GJA1   2267  TATGGTGTTCAAATTTGAACCTTTCTCATGGATTTTTGTGGTGTGGGCCAATATGGTGTT  2326
             ||  ||||||||||||||||||||||||||||||||||||||||||||||||| ||||||
GJA1P1 2270  TACAGTGTTCAAATTTGAACCTTTCTCATGGATTTTTGTGGTGTGGGCCAATACGGTGTT  2329

GJA1   2327  TACATTATATAATTCCTGCTGTGGCAAGTAAAGCACACTTTTTTTTT-CTCCTAAAATGT  2385
             |||||||||||||||||||||||||||||||| |||||||||||||| ||||||||||||
GJA1P1 2330  TACATTATATAATTCCTGCTGTGGCAAGTAAAACACACTTTTTTTTTTCTCCTAAAATGT  2389

GJA1   2386  TTTTCCCTGTGTATCCTATTATGGATACTGGTTTTGTTAATTATGATTCTTTATTTTCTC  2445
             ||||||| ||||||||||||| ||||||| ||||||||||||||||||||||||||||||
GJA1P1 2390  TTTTCCCCGTGTATCCTATTACGGATACTAGTTTTGTTAATTATGATTCTTTATTTTCTC  2449

GJA1   2446  TCCTTTTTTTAGGATATAGCAGTAATGCTATTACTGAAATGAATTTCCTTTTTCTGAAAT  2505
             || |||||||||||||||||||||||||||||||||||||||||||||||| ||||||||
GJA1P1 2450  TCTTTTTTTTAGGATATAGCAGTAATGCTATTACTGAAATGAATTTCCTTTCTCTGAAAT  2509

GJA1   2506  GTAATCATTGATGCTTGAATGATAGAATTTTAGTACTGTAAACAGGCTTTAGTCATTAAT  2565
             ||||||| |||||||||||||| |||||||||||||||||||||||||||||||||||||
GJA1P1 2510  GTAATCACTGATGCTTGAATGACAGAATTTTAGTACTGTAAACAGGCTTTAGTCATTAAT  2569

GJA1   2566  GTGAGAGACTTAGAAAAAATGCTTAGAGTGGA-----CTATTAAATGTGCCTAAATGAAT  2620
             ||||||||||||||||||||||||||||||||     |||||||| ||||||||||||||
GJA1P1 2570  GTGAGAGACTTAGAAAAAATGCTTAGAGTGGAGTGGACTATTAAACGTGCCTAAATGAAT  2629

GJA1   2621  TTTGCAGTAACTGGTATTCTTGGGTTTTCC-TACTTAATACACAGTAATTCAGAACTTGT  2679
             ||||||||||||||||||||||||||| || |||||||||||||||||||||||||||||
GJA1P1 2630  TTTGCAGTAACTGGTATTCTTGGGTTTGCCCTACTTAATACACAGTAATTCAGAACTTGT  2689

GJA1   2680  ATTCTATTATGAGTTTAGCAGTCTTTTGGAGTGACCAGCAACTTTGATGTTTGCACTAAG  2739
             |||||     ||||||| ||||||||||||||||||||||||||||||||||||||||||
GJA1P1 2690  ATTCT-----GAGTTTAACAGTCTTTTGGAGTGACCAGCAACTTTGATGTTTGCACTAAG  2744

GJA1   2740  ATTTATTTGGAATGCAAGAGAGGTTGAAAGAGGATTCAGTAGTACACATACAACTAATT  2799
             |||| |||||||||||||||||||||||||||||||||||||||||| ||||||||
GJA1P1 2745  ATTTCATTTGGAATGCAAGAGAGGTTGAAAGAGGATTCAGTAGTACACATATAACTAATT  2804

GJA1   2800  TATTTGAACTATATGTTGAAGACATCTACCAGTTTCTCCAAATGCCTTTTTTAAAACTCA  2859
             ||||||||||||||| ||||||||||||||||||||||||||||||||||||||||||||
GJA1P1 2805  TATTTGAACTATATGCTGAAGACATCTACCAGTTTCTCCAAATGCCTTTTTTAAAACTCA  2864

GJA1   2860  TCACAGAAGATTGGTGAAAATGCTGAGTATGACACTTTTCTTCTTGCATGCATGTCAGCT  2919
             ||||||| || |||||||||||||||||||||||||||||| ||||||||    ||||||
GJA1P1 2865  TCACAGACGACTGGTGAAAATGCTGAGTATGACACTTTTCTACTTGCATG----TCAGCT  2920
```

```
GJA1   2920  ACATAAACAGTTTTGTACAATGAAAATTACTAATTTGTTTGACATTCCATGTTAAACTAC  2979
             ||||||||||||||||||||||||||||||||||||||||||||||||||||||||||||
GJA1P1 2921  ACATAAACAGTTTTGTACAATGAAAATTACTAATTTGTTTGACATTCCATGTTAAACTAT  2980

GJA1   2980  GGTCATGTTCAGCTTCATTGCATGTAATGTAGACCTAGTCCATCAGATCATGTGTTCTGG  3039
             |||||||||||||||||| ||||||||||||||||||| ||||||||||||||||||||||||
GJA1P1 2981  GGTCATGTTCAGCTTCACTGCATGTAATGTAGACCTTGTCCATCAGATCATGTGTTCTGG  3040

GJA1   3040  AGAGTGTTCTTTATTCAATAAAGTTTTAATTTAGTATAAACATA  3083
             ||||||||||||||||||||  ||||||||||||| ||
GJA1P1 3041  AGAGTGTTCTTTATTCAATA--GTTTTAATTTAGCAT         3075
```
