# Supplementary material for: Confirmation of Connexin45 Underlying Weak Gap Junctional Intercellular Coupling in HeLa Cells
Source: Biomolecules. 2020 Sep 29;10(10):1389. doi: 10.3390/biom10101389 (PMC7650549; doi:10.3390/biom10101389)
Supplement: Supplementary file 1 [file biomolecules-10-01389-s001.zip › File S.zip!/File S6.pdf]

File S6. Potential off-target loci for gRNAs used in this study. Potential off-target sites were found using Cas-OFFinder (http://www.rgenome.net/cas-offinder/), with following selections: SpCas9 and 5'-NRG-4' for the PAM type, 2 for mismatch number, and Homo sapiens (hg19) for the target genome. Lowercase letters denote mismatched residues.

Target gene	gRNA-PAM sequence	Found sequence	Mismatches	Chromosome	Position (hg19)	Locus	On/Off-target
GJB2	CCTCCTTTGCAGCC ACAACGNRG	CCTCCTTTGCAGCC ACAACGAGG	0	chr13	20763593	*GJB2* , exon	On-target
GJB2	CCTCCTTTGCAGCC ACAACGNRG	CCTCCTTTGCtGCCA CAAgGTGG	2	chr6	41659304	*TFEB* , intron	Off-target
GJB2	CCTCCTTTGCAGCC ACAACGNRG	CCTCCTTTGCAGCC ACAtCtTGG	2	chr5	149818830	Intergenic (between *CD74* and *RPS14*)	Off-target
GJB5	GGGCGCCTCTACCT GAACCCNGG	GGGCGCCTCTACCT GAACCCCGG	0	chr1	35223270	*GJB5* , exon	On-target
GJB5	GGGCGCCTCTACCT GAACCCNGG	GGaCGCCTCTtCCTG AACCCTGG	2	chr6	106346458	Intergenic (between *PREP* and *PRDM1*)	Off-target
GJA1	TTTTCTCCGTGGGG CGAGAGNRG	TTTTCTCCGTGGGG CGAGAGAGG	0	chr6	121768589	*GJA1* , exon	On-target
GJA1	TTTTCTCCGTGGGG CGAGAGNRG	TTTTCTCCaTGGGG CaAGAGAGG	2	chr5	108387542	*FER* , intron	Off-target
GJC1	ATAGCCCAGGTACA TCACAGNRG	ATAGCCCAGGTACA TCACAGAGG	0	chr17	42882903	*GJC1* , exon	On-target
