# Supplementary material for: Confirmation of Connexin45 Underlying Weak Gap Junctional Intercellular Coupling in HeLa Cells
Source: Biomolecules. 2020 Sep 29;10(10):1389. doi: 10.3390/biom10101389 (PMC7650549; doi:10.3390/biom10101389)
Supplement: Supplementary file 1 [file biomolecules-10-01389-s001.zip › File S.zip!/File S7.pdf]

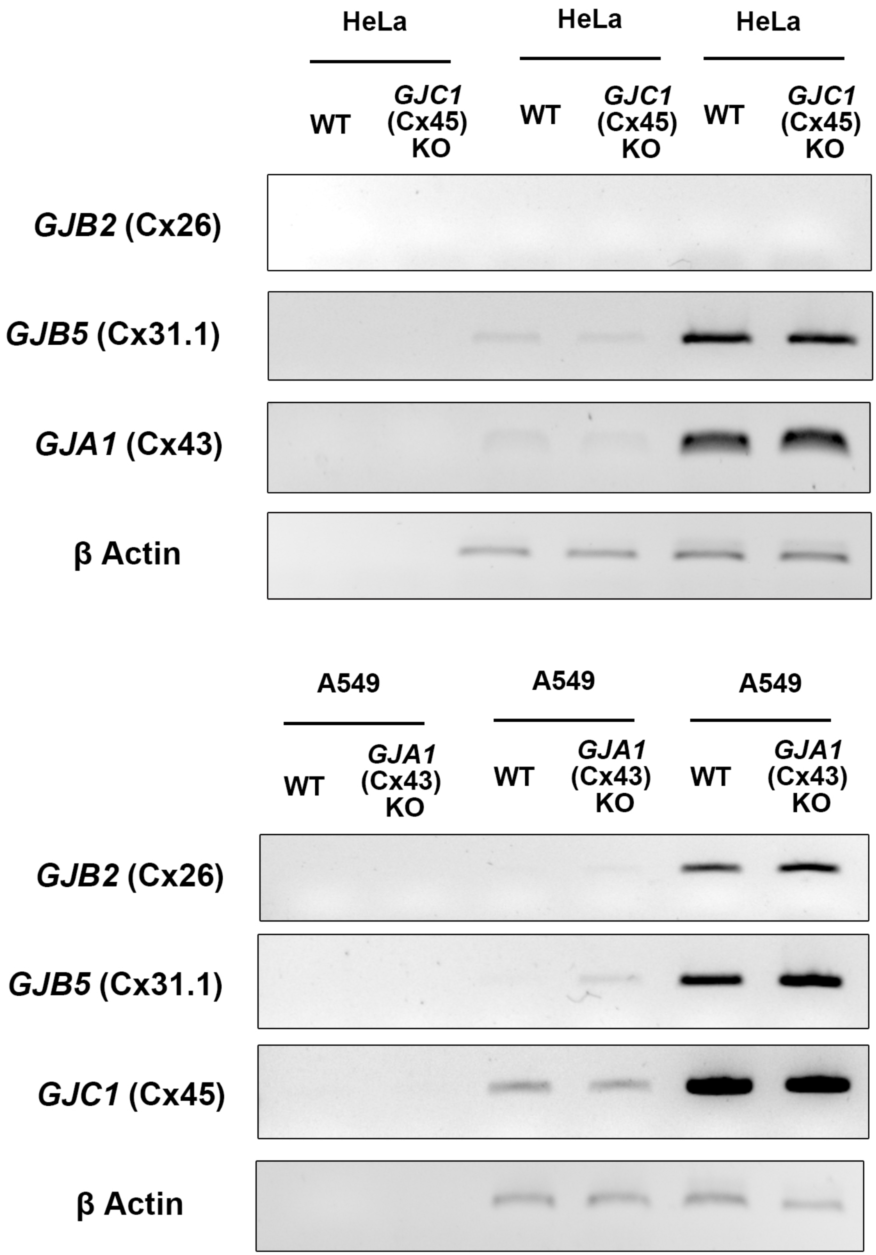

File S7. RT-PCR results at different PCR cycles. Cx genes were PCR-amplified with 25 (lane 1 and 2), 30 (lane 3 and 4), and 35 (lane 5 and 6) cycles. Beta actin was amplified with 15 (lane 1 and 2), 20 (lane 3 and 4), and 25 (lane 5 and 6) cycles.
